# Prevalence, risk factors and adverse pregnancy outcomes of second trimester bacterial vaginosis among pregnant women in Bukavu, Democratic Republic of the Congo

**DOI:** 10.1371/journal.pone.0257939

**Published:** 2021-10-25

**Authors:** Guy Mulinganya, Annelies De Vulder, Ghislain Bisimwa, Jerina Boelens, Geert Claeys, Karen De Keyser, Daniel De Vos, Erick Hendwa, Freddy Kampara, Yvette Kujirakwinja, Jules Mongane, Innocent Mubalama, Mario Vaneechoutte, Steven Callens, Piet Cools

**Affiliations:** 1 Faculty of Medicine, Catholic University of Bukavu, Bukavu, Democratic Republic of The Congo; 2 Department of Obstetrics and Gynecology, Hôpital Provincial Général de Référence de Bukavu, Bukavu, Democratic Republic of The Congo; 3 Department of Internal Medicine and Pediatrics, Faculty of Medicine and Health Sciences, Ghent University, Ghent, Belgium; 4 Department of Diagnostic Sciences, Faculty of Medicine and Health Sciences, Ghent University, Ghent, Belgium; 5 Department of Medical Microbiology, Ghent University Hospital, Ghent, Belgium; 6 Laboratory for Molecular and Cellular Technology, Burn Wound Center, Queen Astrid Military Hospital, Brussels, Belgium; Queen’s University at Kingston, CANADA

## Abstract

**Background:**

Bacterial vaginosis (BV) is the most common gynecological condition in women of reproductive age and associated with adverse pregnancy outcomes. In the Democratic Republic of the Congo (DRC), neonatal mortality rate is as high as 2.8 percent with preterm birth (PTB) and low birth weight (LBW) as leading causes. Because no studies have addressed BV in DRC, we aimed to investigate the prevalence of BV, the risk factors and the association between BV and adverse pregnancy outcomes in a population of pregnant women from Bukavu, DRC.

**Methods:**

A total of 533 pregnant women in the second trimester of pregnancy were recruited in the Provincial Reference Hospital of Bukavu, DRC, between January and October 2017, and followed until delivery. Clinical and sociodemographic data of mother and newborn, and data on (vaginal) hygiene practices, sexual behavior and reproductive history were collected. BV was diagnosed by Nugent scoring of Gram-stained vaginal smears. Two multivariate regression models were built to identify risk factors for BV and to investigate BV as a risk factor for adverse pregnancy outcomes.

**Results:**

The prevalence of BV was 26.3% and approximately half of the women with BV were asymptomatic. Independent risk factors for BV were the use of alternatives to water for intravaginal washing, concurrent partners, unemployed status, the presence of vaginal *Candida* and clay consumption. BV was independently associated with both LBW and PTB of an infant with LBW.

**Conclusion:**

The prevalence of BV in Bukavu is high but in line with the global average. BV was associated with adverse pregnancy outcomes in our study population. Hence, research on modifiable risk factor-based interventions to reduce the prevalence of BV, and on screening/treatment of BV during antenatal care should be explored to reduce neonatal mortality and morbidity.

## Introduction

Bacterial vaginosis (BV) is the most common gynecological disorder in women of childbearing age worldwide [1]. A recent review estimated that the prevalence of BV in the general population in sub-Saharan Africa (SSA) is approximately 25%, in line with the estimated global prevalence (26%) [2]. Microbiologically, BV is understood as a dysbiosis of the healthy vaginal microbiota. This dysbiosis is characterized by the depletion of the one or few *Lactobacillus* species that normally predominate this niche and the heavy overgrowth of a more diverse anaerobic microbiota [3]. BV has been shown to be asymptomatic in the majority of cases (roughly 80% in populations from the United States) [4, 5] but can also be accompanied with clinical symptoms such as discharge, odour, pain, itching and/or a burning sensation. There is a vast literature on sequelae associated with BV including pelvic inflammatory disease [6] and an increased acquisition and/or transmission of sexually transmitted infections such as HIV [7, 8], HSV-2 [9], *Chlamydia trachomatis* [10], *Neisseria gonorrhoeae* [10] and *Trichomonas vaginalis* [10].

Importantly, BV has also been associated with infertility and adverse pregnancy outcomes (APOs) such as chorioamnionitis [11], late miscarriage, spontaneous abortion [12], preterm birth (PTB) and low birth weight (LBW) [13–15]. PTB complications are the leading cause of the yearly 2.9 million neonatal deaths [16]. LBW is an important public health marker and problem [17]. More than 80% of neonatal deaths are newborns with LBW, of which two thirds are born preterm and one third are born term but are small-for-gestational-age [17]. Most of the PTB and LBW burden is in low-income settings, with SSA having the highest PTB rates [16–18]. One of the countries with the highest neonatal mortality rates is the Democratic Republic of the Congo (DRC) (14‰) [19]. However, data on the prevalence of BV and risk factors for BV are largely unknown in SSA and–to the best of our knowledge–non-existing for DRC. Furthermore, no studies in DRC, and only very few in SSA, have investigated the association of BV with adverse pregnancy outcomes. Therefore, the aim of this study was to examine the prevalence of BV, the associated risk factors and adverse pregnancy outcomes in women from Bukavu, DRC.

## Materials and methods

### Ethical approval

This study was ethically approved by the internal review board of the Catholic University of Bukavu (reference number UCB/CIE/NC/016/2016), by the Ministry of Public Health of DRC (reference number 062/CD/DPS/SK/2017) and by the Ethical Committee of Ghent University Hospital (reference number PA2014/003). Women who were found eligible for the study were orally informed in detail on the study and were asked to sign an informed consent form.

### Study design, population and context of the study

The current study is part of the AVEONS study, an acronym of Angamiza Vizuri (Swahili for “break well” or “stop”) Early Onset Neonatal Sepsis. The AVEONS study has the overall aim to describe the prevalence of early onset neonatal sepsis (EONS) in Bukavu (South Kivu, DRC), to document the pathogens causing EONS and their antibiotic susceptibility patterns, and to investigate the role of maternal infections during pregnancy on APOs.

The province of South Kivu is located in the eastern part of DRC, a region characterized by high social, economic and political instability [20, 21]. Thousands of people have been forced to flee their homes and seek refuge either in larger cities such as Bukavu or in neighboring countries (Tanzania and Burundi) [22, 23]. In South Kivu, the prevalence of contraception use by married women and unmarried sexually active women has been estimated to be respectively 64.8% and 94.5% [19]. The fertility rate in South Kivu has been appraised at 7.6%, the access to at least one antenatal visit for pregnant women 93.5% and the home delivery rate 8.9% [19].

The AVEONS study was a prospective observational study where pregnant women were seen between 16 and 20 weeks (visit 1 (V1) = recruitment visit), between 36 and 38 weeks (V2) and at delivery. Newborns were seen at delivery and during the first week of life. This first manuscript of the AVEONS study reports on a cross sectional analysis of women seen at V1 and pregnancy outcomes.

### Recruitment and inclusion in the study

We recruited study participants among pregnant women seeking antenatal care at the Provincial Referral Hospital of Bukavu (PRHB) between January and October 2017. To increase awareness of the AVEONS study, church announcements, radio and TV spots and posters were used. Women were also informed on the study during community leaders’ meetings. Pregnant women who were interested in participating were briefed individually in detail. If women were still interested after this information session, they were screened for inclusion applying the inclusion and exclusion criteria. Women were considered for inclusion if they were between 16–20 weeks of gestational age, agreed to receive antenatal care only at PRHB, accepted to deliver at PRHB and were willing to be contacted by phone (to increase adherence by phone call and text message reminders). Women were not considered eligible if they planned to move out of Bukavu during their pregnancy, had twin pregnancies, suffered from genital bleeding and/or if they used antibiotics in the two weeks before V1. Pregnant women were reimbursed for their transport to and from PRHB.

### Routine antenatal care and delivery procedures

At V1, a general physical examination including anthropometric measurements (including height, weight, body mass index (BMI) and mid-upper arm circumference (MUAC)) was performed. A gynecological examination was performed, and the vaginal mucosa and cervix were inspected for sores and tumors. In case women had abnormal vaginal discharge, an abnormal foul smell, experienced itching and/or a burning sensation after intercourse, a vaginal infection was diagnosed according to the syndromic-based protocol for the management of pregnancy issued by the Ministry of Public Health of DRC [24]. These symptomatic women were treated empirically according to the local protocol (daily clotrimazole (200 mg) against candidiasis and clindamycin (100 mg) against BV for six days). In case of allergy against clindamycin, a daily metronidazole ovule was prescribed for six days.

During the gynecological examination, the vaginal pH was determined by means of an indicator pH paper (Hilindicator pH paper). An ultrasound examination was performed at V1 to assess the viability of the fetus and the cervical length. At 24 weeks of gestation, participants were offered a single dose of mebendazole (500 mg) against soil-transmitted helminths and a single dose of sulfadiazine-pyrimethamine (500 mg) against malaria as recommended in the pregnancy protocol. Participants further followed antenatal care as usual.

At V1, five mL of blood was collected in a tube without anticoagulant. Subsequently, the serum was tested for HIV, malaria and hemoglobin using rapid tests (Alere Determine^™^ HIV-1/2 (Abbott), Malaria AG P.f/pan (Bioline) and Hemocue Hb201+ (Hemocue AB), respectively). A urine sample was collected in a sterile container and tested for the presence of white blood cells and nitrite (indicating a urinary tract infection or bacteriuria) by Multistix^®^ dipsticks (Siemens). A vaginal swab (COPAN) was taken by gently rolling the top of the swab against the midportion of the vaginal wall. The swab was rolled on three glass slides, one smear was used for routine wet mount microscopy and two were used for Gram-staining (Nugent score). The wet mount was examined microscopically immediately after collection of the vaginal swab for the presence of clue cells (one of the four Amsel criteria, used for the diagnosis of BV in clinical practice), *Candida* cells and/or hyphae and *Trichomonas vaginalis*.

At delivery, the labor was followed, and appropriate data such as obstetrical parameters (uterine height, fetal presentation, status of the fetal membranes, number of vaginal examinations, maternal temperature, the use of the childbirth kit) were collected by nurses and the senior assistant. Neonates were subjected to a general examination and anthropometric parameters (length, weight and head circumference) were assessed by a pediatrician. The presence of an early neonatal infection was assessed based on the WHO criteria, i.e., the presence of at least one of the following signs: temperature instability, lethargy, feeding intolerance, respiratory distress, hemodynamic instability, convulsion, hypotonia, irritability or bleeding diathesis [25].

### Study specific laboratory tests

Slides for Gram-staining were heat fixated, stored at room temperature and shipped to the Laboratory Bacteriology Research (Ghent University, Ghent, Belgium), where Gram-staining was carried out with an automated PolyStainer (IUL). All Gram-stained slides were scored according to the Nugent scoring system [26] for the laboratory-based diagnosis of BV. Briefly, five microscopic fields per Gram-stained slide were scored on a 1000x magnification for the presence and quantity of *Lactobacillus* (Gram-positive rods), *Gardnerella vaginalis*/*Bacteroides* (Gram-variable coccobacilli) and *Mobiluncus* (Gram-negative curved rods) cell types. The smear was then categorized as representing a healthy vaginal microbiota (Nugent score 0–3), an intermediate vaginal microbiota (Nugent score 4–6) or BV (Nugent score 7–10). Furthermore, during the Nugent scoring, we also documented the presence of *Candida* cells and/or hyphae. All slides were scored single-blinded by two independent readers. In case of a discrepancy in categorization, the slide was reassessed by the two reviewers and discussed. If no consensus was obtained, a third person assessed the slide as a tie breaker.

### Questionnaires

At V1, data on the sociodemographic characteristics, reproductive health history, sexual behavior, vaginal practices and complaints of the pregnant women were obtained in a confidential way using a questionnaire (S1 File). Women were questioned individually in Swahili by a gynecologist or gynecologist in training, who was also responsible for the patient’s care. The interview took about 20 minutes and answers were noted on printed questionnaires.

### Data analysis

All clinical data forms, laboratory forms and questionnaires of each participant were scanned and stored as a single file. The raw data were then captured in the CSPRO software by means of double data entry. The ‘compare data tool’ of the CSPRO software was used to compare the two data sets. In case of discrepancies, the raw data were consulted, and discrepancies were solved. The two datasets were further checked for discrepancies in STATA 14 (Stata Corp, College Station, Texas, USA) before making one final locked dataset for analysis.

Categorical variables were summarised into frequencies and proportions, continuous variables into median and interquartile range (IQR).

First, we determined the prevalence of BV, defined based the Nugent score, and reported this prevalence with a 95% confidence interval (CI). A Kappa test was calculated to evaluate the agreement in categorization based on the Nugent score between the two readers. The agreement was considered as poor (<0.00), slight (0.00–0.20), fair (0.21–0.40), moderate (0.41–0.60), substantial (0.61–0.80) or almost perfect (0.81–1.00) [27].

Second, to determine independent risk factors for BV (defined as a Nugent score of 7–10), we built a multivariate model. Here, plausible explanatory variables were sociodemographic characteristics, sexual behavioral and vaginal hygiene characteristics and laboratory findings. Logistic regression was applied to assess the association between the various risk factors and BV. Independent variables with a p-value <0.10 in the univariate analysis were incorporated into a multiple regression model. In this model, independent variables were considered as statistically significantly associated with BV if the p-value was <0.05.

Third, to determine whether BV itself was a risk factor for one or several APOs, we estimated unadjusted odds ratios and adjusted odds ratios applying logistic regression models. We considered PTB, LBW, preterm birth of a LBW infant (PTB-LBW), premature rupture of membranes (PROM), and suspicion of EONS as dependent variables. Here, BV was considered as the independent variable. PTB was defined as delivery before 37 completed weeks of gestation, LBW as birth weight <2,500 g at delivery, PTB-LBW as delivery occurring before 37 weeks of gestation with a birth weight < 2,500 g. Suspicion of EONS was defined on the basis of WHO criteria, i.e., a neonate with minimum one of the following symptoms: temperature instability, lethargy, feeding intolerance, respiratory distress, hemodynamic instability, convulsion, hypotonia, irritability or bleeding diathesis [25]. We did not assess the association between BV and second trimester pregnancy loss because the number of cases were not sufficient to draw statistically significant conclusions. A modified Poisson regression model with robust standard error was built using generalized linear regression equations to model the link. APOs with a significant association with BV in the univariate analysis were selected for the multivariate model. In this model, to better control the effect of BV on APOs, we included a priori covariates for APOs that were expected to be relevant, based on literature [15, 28–30]. These covariates were vaginal *Candida* colonization (as assessed by means of microscopic examination of Gram-stained slides), anemia, MUAC, cervix length, parity, BMI, maternal age, education level, previous PTB and high diastolic blood pressure at V1 (≥ 90 mm Hg).

## Results

### Sociodemographic description of the study population

**Fig 1:**
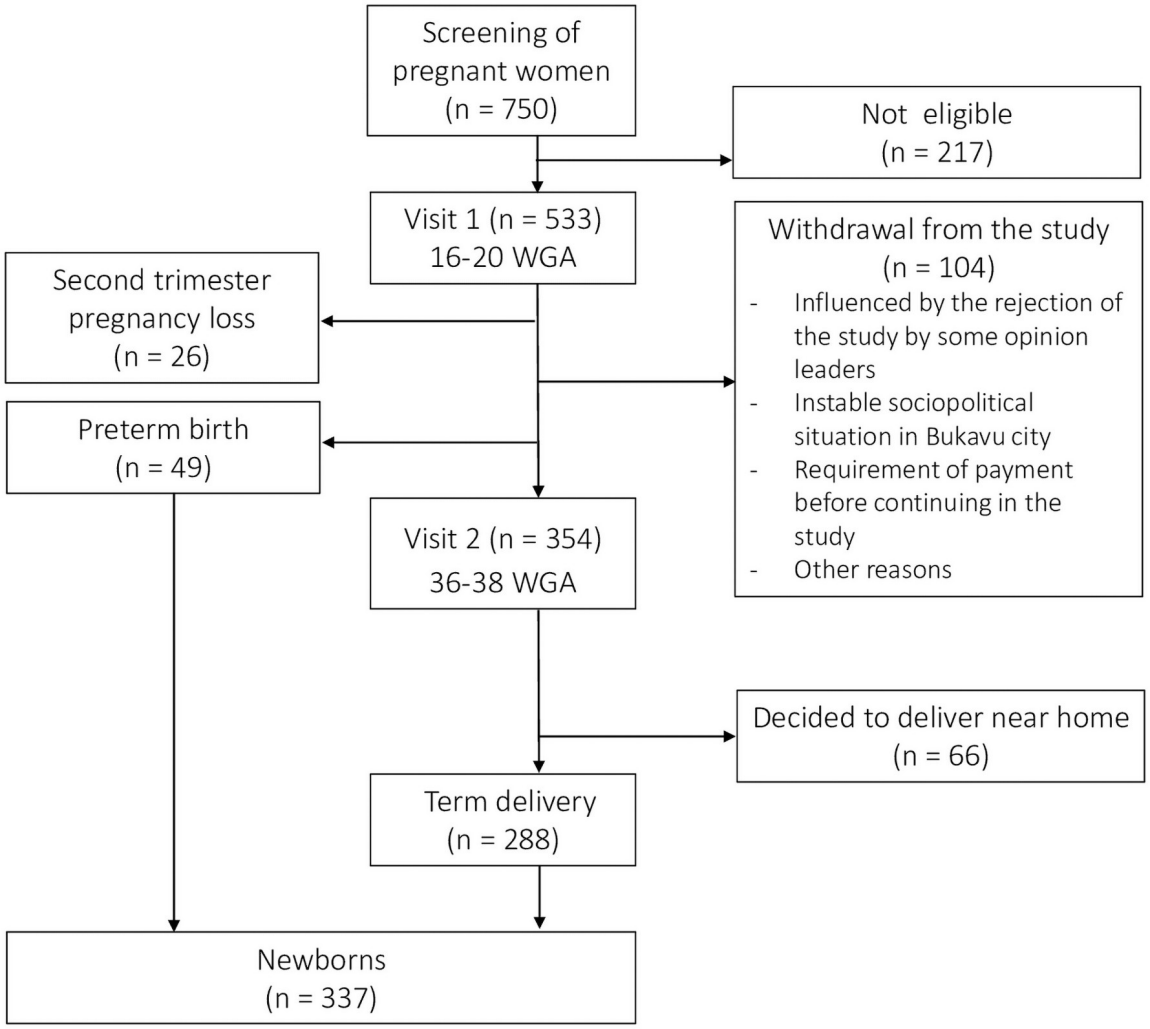
Flowchart of study. WGA, weeks of gestational age.

The flowchart of the number of pregnant women that were screened, included in the study and seen over the different visits and the number of newborns is shown in Fig 1.

A total of 750 pregnant women were screened, and 533 were found eligible and enrolled in the cohort (V1). A total of 104 women (19.5%) who started in the study withdrew from the study before V2. The main reasons were the rejection of the study by some opinion leaders (who incorrectly thought women received a substantial payment for participation) and the unstable sociopolitical situation in Bukavu city during the study period. A total of 26 women had a second trimester pregnancy loss. Of the 354 women who completed V2, 66 (12.4%) decided to not deliver at PRHB but near their home and 288 delivered at term. Of all newborns in the study, 288 (85.5%) were born at term and 49 (14.5%) were born preterm. Data for seven newborns were unavailable during follow-up due to uncompleted forms.

The sociodemographic characteristics of the study population are summarized in Table 1 and presented in SI2. The median age was 28.0 years (IQR 8.0 years). All participants lived in Bukavu. The majority lived in poverty (72.6%), completed the primary school (88.4%) and nearly all were married (95.5%). Most participants were from the Shi tribe (66.7%) and almost all were Christians (93.7%).

**Table 1:** Sociodemographic characteristics and pregnancy outcomes of the study population stratified by vaginal microbiota categorization.

Characteristics of pregnant women	Number 525^1^ (%)	Vaginal microbiota		
		Healthy microbiota 285 (54.3)	Intermediate microbiota 102 (19.4)	Bacterial vaginosis. 138 (26.3)
**Age at recruitment**				
<20 years	26 (5.0)	13 (4.6)	5 (4.9)	8 (5.8)
20–24 years	113 (21.5)	65 (22.8)	17 (16.7)	31 (22.5)
25–29 years	172 (33)	94 (33.0)	36 (35.3)	43 (31.2)
30–34 years	135 (25.7)	72 (25.3)	30 (29.4)	33 (23.9)
≥35 years	78 (14.9)	41 (14.4)	14 (13.7)	23 (16.7)
**Tribe**				
Shi	374 (71.2)	203 (71.2)	83 (81.4)	88 (63.8)
Rega	57 (10.9)	32 (11.2)	8 (7.8)	17 (12.3)
Other tribes^2^	94 (17.9)	50 (17.5)	11 (10.8)	33 (23.9)
**Religion** ^3^				
Christians	492 (93.7)	266 (93.3)	94 (92.2)	132 (95.7)
Not Christian	33 (6.3)	19 (6.7)	8 (7.8)	6 (4.4)
**Educational level** ^4^				
Primary	62 (11.8)	35 (12.3)	11 (10.8)	16 (11.6)
Secondary	273 (52.0)	151 (53.0)	54 (52.9)	68 (49.3)
Higher	190 (36.2)	99 (34.7)	37 (36.3)	54 (39.1)
**Quality of life** ^5^				
Poor	381 (72.6)	209 (73.3)	74 (72.6)	98 (71.0)
Not poor	144 (27.4)	76 (26.7)	28 (27.5)	40 (29.0)
**Employment status**				
Employed or self-employed	89 (17.0)	57 (20.0)	17 (16.7)	15 (10.9)
Unemployed	436 (83.1)	228 (80.0)	85 (83.3)	122 (89.1)
**Marital status**				
Married	501 (95.4)	273 (95.8)	100 (98.0)	128 (92.8)
Unmarried	24 (4.6)	12 (4.2)	2 (2.0)	10 (7.3)
**Clinical status**^6^ **at first visit**				
Symptomatic	254 (48.4)	126 (44.2)	55 (53.9)	73 (52.9)
Asymptomatic	271 (51.6)	159 (55.8)	47 (46.1)	65 (47.1)

^1^ Eight slides did not contain biological material or appeared damaged,

^2^ Tembo, Fuliru, Hunde, Nyanga, Hutu, Nande, Vira, Bembe, each with a proportion < 2.5%;

^3^ Christian represents Catholics, Protestants, Anglicans, Kimbanguistes and Christian Revival Church; not Christian represents Muslims, Animists, and atheists;

^4^ Participants who not yet ended their level were included in that level;

^5^ Taking into account local parameters, poverty was calculated considering the type of the floor, water source, electricity and commodities in the house. The total score ranged from 4 to 17, a score < 10 was considered under the threshold of poverty, a score ≥10 was considered above the threshold of poverty. We did not include income in the score calculation because it is very unstable and depends mainly on the informal sector,

^6^ Participants were symptomatic if they presented with abnormal vaginal discharge, vaginal itching, burning vaginal sensation after sexual intercourse and/or foul smell from vagina.

The pregnancy outcomes of the study population are summarized in Table 2.

**Table 2:** Pregnancy outcomes stratified by vaginal microbiota categorization.

Outcomes	Number 330^1^ (%)	Vaginal microbiota		
		Healthy microbiota 182 (55.1)	Intermediate microbiota 64 (19.4)	Bacterial Vaginosis 84 (25.5)
**Low birth weight** ^2^				
Yes	17 (5.2)	5 (2.8)	4 (6.3)	8 (9.5)
No	313 (94.9)	177 (97.3)	60 (93.8)	76 (90.5)
**PROM** ^3^				
Ruptured	68 (20.6)	35 (19.2)	13 (20.3)	20 (23.8)
Intact	262 (79.4)	147 (80.8)	51 (79.7)	64 (76.2)
**Admission to NICU for suspicion of early-onset neonatal sepsis**				
Yes	14 (4.2)	10 (5.5)	3 (4.7)	1 (1.2)
No	316 (95.8)	172 (94.5)	61 (95.3)	83 (98.8)
**Preterm birth** ^4^				
Yes	48 (14.6)	25 (13.7)	9 (14.1)	14 (16.7)
No	282 (85.5)	157 (86.3)	55 (85.9)	70 (83.3)
**Preterm delivery of low birth weight**				
<2500gr	15 (31.3)	4 (16.0)	4 (44.4)	7 (50.0)
≥2500gr	33 (68.8)	21 (84.0)	5 (55.6)	7 (50.0)

NICU, neonatal intensive care unit; PROM, premature rupture of membranes;

^1^ Data for seven newborns were unavailable during follow-up due to uncompleted forms;

^2^ <2500 gram [31];

^3^ Premature rupture of membranes before the onset of the labor;

^4^ <37 completed weeks of gestation [31].

### The prevalence of BV

A total of 525 slides were scored according to Nugent. Eight slides did not contain biological material or appeared damaged. The Kappa statistic of the interrater agreement between the two independent readers after the first reading was 0.84 (considered *almost perfec*t [27]) and all discrepancies were resolved. A total of 285 women (54.3%; 95% CI, 49.9–58.6%) had a healthy vaginal microbiota, whereas 102 (19.4%; 95 CI, 16.1–23.1%) had an intermediate microbiota and 138 (26.3%; 95% CI, 22.6–30.3%) had BV. Slightly less than the half of the pregnant women with BV were asymptomatic (47.1%).

### Risk factors for BV

The univariate associations are shown in SI3. Six risk factors were found to be significantly associated with BV at the p<0.05 level. The highest OR for BV was found to be (an) extramarital relationship(s) of the study participant (OR: 4.35; 95% CI: 1.21–15.66). One or more extramarital partner of the husband were also associated with higher odds of BV (OR: 2.34; 95% CI: 1.29–4.23). The odds of having BV was approximately twice as high for women with vaginal *Candida* (as assessed by microscopic examination of Gram-stain smears) (OR: 1.60; 95% CI: 1.06–2.43). Women who were unemployed were more likely to have BV (OR: 1.94; 95% CI: 1.07–3.51). Clay consumption was associated with lower odds of BV (OR 0.58; 95% CI: 0.36–0.94). Women who used varied substances for intimate toilets and women who were from minority tribes also had higher odds of having BV (OR: 2.03; 95% CI: 1.28–3.23 and OR: 1.76; 95% CI: 1.08–2.86).

The multivariate model for risk factors of BV is shown in Table 3. In this model, extramarital partner(s) of the husband was found to be an independent risk factor for BV (aOR: 2.15; 95% CI: 1.14–4.05). Also, the use of varied substances (compared to water) during intimate toilets was found to be independently associated with BV (aOR: 2.13; 95% CI:1.30–3.49). The detection of *Candida* cells and/or hyphae in Gram-stained vaginal smears was an independent risk factor for BV (aOR: 1.59; 95% CI: 1.03–2.46). Women who consumed clay had lower odds of having BV (aOR: 0.47; 95% CI: 0.28–0.78). The unemployed status was found to be independently associated with BV (aOR: 1.95; 95% CI: 1.06–3.61) and more than one sexual partner (of the participant) during lifetime was independently associated to BV (aOR: 1.55; 95% CI: 1.02–2.34).

**Table 3:** Multivariable model of risk factors associated with bacterial vaginosis in pregnancy.

Variable	aOR (95% CI)
Unemployed status	**1.95 (1.06–3.61)**
Husband has had concurrent extra-marital female sexual partners in the last six months	**2.15 (1.14–4.05)**
Pregnant woman has had concurrent extra-marital male sexual partners in the last six months	3.54 (0.83–15.03)
>1 sexual partner (of the participant) during lifetime	**1.55 (1.02–2.34)**
Substances (other than water) used during intimate toilet^1^	**2.13 (1.30–3.49)**
Clay consumption^2^	**0.47 (0.28–0.78)**
Vaginal *Candida* cells and/or hyphae on Gram-stained vaginal smears	**1.59 (1.03–2.46)**

aOR, adjusted odds ratio; CI, confidence interval;

^1^ Soap, herbs, mixed powders, lemon, disinfectant products;

^2^ More or equal to a regular total consumption of 100 g of clay per day during current pregnancy; Bold, p<0.05.

### BV as risk factor for adverse pregnancy outcomes

Table 4 provides the univariate associations of BV and an intermediate vaginal microbiota with APOs. BV was statistically significantly associated with higher risk of LBW (RR: 3.47; 95% CI: 1.17–10.30) and LBW for PTB (RR: 3.12; 95% CI: 1.09–8.94). No statistically significant associations were found between BV and the other APOs (PTB, PROM, suspicion of EONS), and between an intermediate vaginal microbiota and any of the APOs.

**Table 4:** Univariate analysis of BV and an intermediate vaginal microbiota as risk factor for adverse pregnancy outcomes.

Outcomes	PTB		LBW		PTB-LBW		PROM		Suspicion of EONS	
	OR (95% CI)	p-value	OR (95% CI)	p-value	OR (95% CI)	p-value	OR (95% CI)	p-value	OR (95% CI)	p-value
Healthy vaginal microbiota	Ref.		Ref.		Ref.		Ref.		Ref.	
Intermediate vaginal microbiota	1.02 (0.50–2.08)	0.948	2.28 (0.63–8.23)	0.210	2.78 (0.86–8.95)	0.087	1.06 (0.60–1.87)	0.851	0.85 (0.24–3.01)	0.805
Bacterial vaginosis	1.21 (0.66–2.22)	0.529	**3.47 (1.17–10.30)**	**0.025**	**3.12 (1.09–8.94)**	**0.034**	1.24 (0.76–2.01)	0.389	0.22 (0.03–1.67)	0.142

CI, confidence interval; EONS, early-onset neonatal sepsis; LBW, low birth weight; OR, Odds ratio; PROM, premature ruptures of membranes; PTB, preterm birth; PTB-LBW, preterm birth of a low birth weight newborn; Ref., Reference category; Bold, p<0.05.

Table 5 documents the multivariable association of BV with those APOs found to be significantly associated in univariate analysis, i.e., PTB and PTB-LBW. In this model, we corrected for different possible confounding factors identified in literature. BV was significantly and independently associated with a higher risk of both LBW (adjusted relative risk (ARR): 3.52; 95% CI: 1.20–10.32) and PTB-LBW (ARR: 5.67; 95% CI: 2.12–15.18).

**Table 5:** Multivariate model for the association between bacterial vaginosis (risk factor) with low birth weight or low birth weight for preterm birth.

	LBW	PTB-LBW
	ARR (95%CI)	ARR (95%CI)
**Vaginal microbiota**		
Intermediate vaginal microbiota	1.64 (0.39–6.87)	**4.57 (1.34–15.57)**
Bacterial vaginosis	**3.52 (1.20–10.32)**	**5.67 (2.12–15.18)**
**Haemoglobin < 110 g/L**	**3.84 (1.55–9.52)**	**5.86 (1.96–17.50)**
**Cervical length(mm) ≥30 mm**	0.41 (0.14–1.16)	1.80 (0.64–5.05)
**Parity ≥3**	**0.18 (0.03–0.99)**	**0.20 (0.08–0.55)**
**Previous preterm birth**	**9.73 (2.77–34.14)**	**7.08 (1.89–26.62)**
**Age at first sexual intercourse ≥ 18**	0.49 (0.19–1.25)	0.59 (0.28–1.25)

Model adjusted for confounder factors (sociodemographic characteristics, sexual behavioral parameters, vaginal hygiene practices and laboratory findings). ARR, adjusted relative risk; bold, p<0.05.

## Discussion

BV is the most prevalent gynecological condition in women of reproductive age worldwide [2] associated with discomfort, sexually transmitted co-infections and APOs, but–to the best of our knowledge–no studies on BV have been performed in DRC.

Our study in pregnant women from Bukavu (DRC) shows a BV prevalence of 26.3%, which is in line with the BV prevalence of 25% in the general population of women of reproductive age in SSA, documented in a recent meta-analysis [2]. Studies that assessed the prevalence of BV in pregnant women from SSA are summarized in Table 6 and report prevalences ranging from 6.4% (Burkina Faso) to 48.5% (Uganda). Among the possible reasons for the discrepancies in prevalence between these different studies are varying criteria of diagnosis of BV (Nugent, Amsel, Wet mount) [32, 33], different behavioral characteristics such as sexual behavior and vaginal hygienic practices [34], but also different ethnical profile and sociodemographic characteristics [35].

**Table 6:** Summary of studies reporting on the prevalence of BV in pregnant women from sub-Saharan Africa.

Country	Prevalence (%)	Asymptomatic (%)	Diagnosis	Reference
Botswana	38.0	76.0	Nugent	[36]
Burkina-Faso	6.4	N/A	Nugent	[37]
Burkina-Faso	13.0	N/A	Amsel^#^	[38]
Ethiopia	19.4	63.3	Nugent	[39]
CAR	29.1	N/A	Nugent	[40]
**DRC**	**26.3**	**47.1**	**Nugent**	**current study**
Kenya	19.3	N/A	Nugent	[41]
Kenya	29.0	N/A	Nugent	[34]
Kenya	22.0	N/A	Nugent	[42]
Malawi	30.0	N/A	Wet mount	[43]
Nigeria	11.9	N/A	Amsel	[44]
Nigeria	26.0	N/A	Nugent	[13]
Nigeria	38.0	N/A	Nugent	[45]
South-Africa	31.0	N/A	Nugent	[34]
South-Africa	37.3	47.1%	Nugent	[46]
Tanzania	28.5	N/A	Nugent	[47]
Tanzania	20.9	N/A	Amsel	[48]
Togo	38.2	N/A	Nugent	[49]
Uganda	48.5	N/A	Nugent	[50]
Zimbabwe	32.6	N/A	Amsel	[51]

^#^detection of clue cells, one of the Amsel criteria, was performed on Gram-stained slides instead of on wet mounts (which is normally used when using Amsel criteria); CAR, Central African Republic; DRC, Democratic Republic of the Congo.

In our study, nearly half of women diagnosed with BV by means of the Nugent score were asymptomatic. Compared to other studies in SSA (Table 6), the proportion of women in our population that was asymptomatic is largely comparable but smaller to what is reported in studies from industrialized countries [52].

### Concurrent sexual partners, vaginal hygienic practices and Candida are independent risk factors for BV

There is controversy regarding BV being a sexually transmitted infection (STI). According to several authors, BV is not an STI because of the lack of a clear etiological agent and the lack of a peer disease in men [53, 54]. Other authors support the hypothesis that BV is a sexually associated or enhanced disease, rather than an STI [55, 56]. We found that pregnant women whose partners had concurrent sexual partners had a two-fold increase in BV compared to pregnant women whose partner did not have other sexual partners. Raising awareness on the potential harm that multiple sex partners may have on a healthy vaginal microbiota–even with condom use [56]–could be a potential way to promote a healthy pregnancy.

We found that women who applied substances intravaginally such as soap, antiseptics, lemon juice or herbs had a nearly two-fold higher chance of having BV. In DRC, these practices are performed for hygienic reasons (soap, antiseptics) and/or to increase sexual pleasure for both men and women by drying and tightening the vagina (lemon juice, herbs, plants, powders) [57]. An individual participant data meta-analysis reported a positive association between disturbances of the healthy vaginal microbiota and intravaginal cleaning with soap, but not with the application of cloth or paper in women from SSA [58]. In contrast, in a multi-country study in SSA, Jespers and coworkers (2014) did not find an association between BV and intravaginal washing, drying or tightening of the vagina [34]. In an *in vitro* study, Aslan and coworkers (2018) showed that commercially available vaginal douching products had a detrimental effect on vaginal lactobacilli [59]. In our study, we learned that most women had no knowledge on BV and that they were convinced that an intimate toilet using varied substances was beneficial for hygienic reasons, although we did not specifically ask about this in our questionnaires.

In our study, concurrent vaginal *Candida* colonization was independently associated with an approximately two-fold increased odds for BV. A study in Chinese pregnant women found that vaginal candidiasis, defined by means of wet mount, was an independent risk factor for BV (defined by the Nugent score) [60]. In India, Rathod and co-workers (2012) found that women clinically diagnosed with BV, but not women with BV defined by Nugent, had a higher prevalence of vulvovaginal candidiasis, defined as a *Candida*-positive vaginal culture with the presence of vaginal signs and symptoms (discharge, itching, erythema) [61]. In pregnant women from Mwanza (Tanzania), Shayo and co-workers did not find an association between BV (defined by Nugent) and candidiasis (methodology of assessment not reported) [47]. Of note, pregnant women have been reported to have a higher prevalence of *Candida* colonization/vaginitis compared to non-pregnant women [34, 62, 63].

In our study, over a quarter of pregnant women reported to consume clay. This geophagia was independently associated with a lower chance of having BV, for reasons that remain unclear to us. Interestingly, several authors have reported that some clays have an *in vitro* bactericidal effect against a wide range of bacteria [64–66], but the antibacterial mechanisms remain unclear [64]. Possibly, a similar bactericidal effect might help to eradicate the BV-associated microbiota. Also, severe BV has been independently and inversely associated with intake of some nutrients such as folate, vitamin E and calcium [67], and it might be possible that such an (improved) uptake is linked to clay geophagia in our study. However, in general, clay consumption should be discouraged because of the several possible harmful effects such as the presence of high iron oxides, high lead concentration, infectious eggs of soil-transmitted helminths, and the association with anemia [68, 69].

### BV is an independent risk factor for low birth weight and preterm delivery of a newborn with low birth weight, but not for PTB

In our study population, compared to women with a healthy vaginal microbiota, pregnant women with BV had approximately a fourfold increased risk to deliver a neonate with LBW and a sixfold increased risk to deliver a preterm neonate with LBW. In a multicentre study from the United States of America, Hillier and co-workers found a similar independent association between BV and PTB-LBW (aOR, 1.4) [29]. A large Danish cohort study also reported BV to be significantly and independently associated with both LBW (aOR, 2.0) and PTB-LBW (aOR 2.5) [70].

Globally, one in seven newborns are born with a LBW. LBW is a crucial underlying determinant and contributor to neonatal and infant mortality and nearly half of all perinatal and one-third of all infant deaths are in newborns with LBW [71]. Given that BV increases the risk for LBW in our study population, further studies should explore whether behavioural changes addressing the BV risk factors we identified can reduce LBW prevalence. For example, women could be informed on the harmful effects of certain vaginal hygienic practices during antenatal care or family planning. BV merits more attention and awareness during prenatal care, although screening of pregnant asymptomatic women for BV, and treatment to improve pregnancy outcomes, remains controversial [72].

Although several studies report an association of BV with PTB [73–75], in our study, BV was not associated with PTB.

### Study limitations

Our study was limited by the fact that the number of women who delivered at our hospital maternity was less than foreseen due to the socio-political situation in Bukavu during the study period. After inclusion of the first visit, approximately one third of the study participants were lost during follow-up, due to unforeseen circumstances such as delivery near home because of worsening crisis and unsafety in the region, and, sadly because of the negative influence of some opinion leaders in the community. As we were aware of such threats, we tried to overcome this burden by asking in advance whether they were willing to deliver in the hospital (inclusion criterion) rather than at home. Furthermore, one third of participants were payed a small fee for transport to the hospital.

A second limitation is that we only analyzed BV in second trimester and not the first and third trimester. Given that the vaginal microbiota is highly dynamic [76], it is unclear what the BV status of pregnant women in our study population in the first and third trimester was. Further longitudinal studies on a large scale are required to assess the relation between BV during pregnancy and the occurrence of APOs.

A third limitation is that, despite the confidentiality during the participant interview, asking about intimate relationships and extramarital sexual partners, could have biased our results because of taboo. In addition, women might have been unaware of (an) extramarital relationship(s).

## Conclusion

In conclusion, BV was highly prevalent in pregnant women in Bukavu, and associated with modifiable risk factors such as vaginal hygiene practices. Given that BV forms an increased risk for important APOs, further local research is needed to better understand this link and to reduce the BV prevalence in pregnancy.

## Supporting information

S1 File Questionnaire. (PDF)

S2 File Sociodemographics, anthropometrics, sexual and hygiene behavioral parameters, clinical and laboratory findings stratified by status of the vaginal microbiota. (DOCX)

S3 File Univariate regression analysis of pregnant women’s sociodemographics, anthropometrics, sexual and hygiene behavioral parameters; and bacterial vaginosis at visit 1. (DOCX)

S4 File STROBE checklist. (DOCX)
