# Supplementary material for: Prevalence, risk factors and adverse pregnancy outcomes of second trimester bacterial vaginosis among pregnant women in Bukavu, Democratic Republic of the Congo
Source: PLoS One. 2021 Oct 25;16(10):e0257939. doi: 10.1371/journal.pone.0257939 (PMC8544863; doi:10.1371/journal.pone.0257939)
Supplement: S1 File — (PDF) [file pone.0257939.s001.pdf]

Recruitment form (16-20 WG)

Date of recrutement : |___|___| / |___|___| / 201

LMP : |___|___| / |___|___||___|___| 201

Identification N°: I___I___I___I___I___I

Name and last name:

Phone N°: / Husband's Phone N°:

Relative's phone N° Closet health center Phone N°:

Address: N°. Area:

NB: These are confidential information and cannot be disclosed to anyone external to the research team

N°	Filters questions	Coding		
Q101	When were you born?<br><br>NB: if no answer, ask for a historical event or a certain era to estimate the year of birth	Month /Year<br>I don't know<br>No answer	[__I__]/[__I___I___I__]<br>888<br>999	
Q102	What is your age?	Age (year)<br>I don't know<br>No answer	[__I__]<br>888<br>999	
Q103	What tribe do you belong to?	Shi<br>Rega<br>Havu<br>Tembo<br>Hunde<br>Nyanga<br>Hutu<br>Nande<br>Vira<br>Fuliru<br>Bembe<br>Other tribes<br>No answer	1<br>2<br>3<br>4<br>5<br>6<br>7<br>8<br>9<br>10<br>11<br>12<br>13	
Q104	What religion do you belong to?	Catholics<br>Protestant<br>Anglican<br>Kimbanguist<br>Muslim<br>No religion<br>Other religions<br>I don't know<br>No answer	1<br>2<br>3<br>4<br>5<br>6<br>7<br>888<br>999	
Q105	Have you been to school?	Yes<br>No<br>I don't know<br>No answer	1<br>2<br>888<br>999	If no answer to this question move to Q108

R

Recruitment form (16-20 WG)

N°	Filter questions	coding		
Q106	What is the highest level of education that you have reached?	Primary (incomplete)	1	
		Primary (complete)	2	
		Secondary (complete)	3	
		Secondary (incomplete)	4	
		High education (complete)	5	
		High education (incomplete)	6	
		No answer	999	
Q107	How long have you been at school?	<12	1	
		<16	2	
		<18	3	
		>18	4	
		I don't know	888	
		No answer	999	
Q108	Which commune are you living in?	KADUTU	1	
		IBANDA	2	
		BAGIRA	3	
		Outside the city	4	
		I don't know	888	
		No answer	999	
Q109	Which type of toilet do you use?	Flushing toilet	1	
		Turkish toilet with flushing water	2	
		Pit toilet	3	
		Bush	4	
		Other ………………………..	5	
		I don't know	888	
		No answer	999	
Q110	What do you use to wipe after bowel?	Toilet tissue	1	
		Water	2	
		Other…………………………	3	
		I don't know	888	
		No answer	999	
Q111	Are you supplied with electricity? if yes, do you have a refrigerator or a television?	No electricity	1	
		Electricity available without refrigerator/TV	2	
		Solar energy	3	
		Electricity available with refrigerator or TV	4	
		Electricity available with refrigerator and TV	5	
		I don't know	888	
		No answer	999	
Q112	Do you use improved drinking-water facilities?	Tap water with in-house facilities	6	
		Tap water with outdoor installation	5	
		Public water source near home	4	
		Public water source at more than one km	3	
		Rainwater	2	
		Other:______________________	1	
		don't know	888	
		No answer	999	

Recruitment form (16-20 WG)

**Q113**	What fuel do you use to heat food?	Electricity<br>Gas<br>Oil<br>Charcoal<br>Fierwood<br>Other: ___________________<br>I don't know<br>No answer	5<br>4<br>3<br>2<br>1<br>0<br>888<br>999	
**Q114**	What kind of paving do you use ?	Tiles<br>Smoothed cement<br>rough cement<br>only aplastic carpet<br>bare floor<br>Other:___________________<br>I don't know<br>No answer	5<br>4<br>3<br>2<br>1<br>0<br>888<br>999	
**Section 2: Now, I will ask you questions about your couple's life, work and family. These data are of course confidential**				
**Q201**	Are you married ?	Never married<br>Married<br>Live separated<br>Divorced<br>Widow<br>I don't know<br>No answer	1<br>2<br>3<br>4<br>5<br>888<br>999	If answer other than 1 to this question move to Q207
**Q202**	At which age did you get married?	Age (in years)<br>I don't know<br>No answer	[__|__]<br>888<br>999	
**Q203**	How long you lived with your husband, relating to this pregnancy?	years<br>month<br>The score of zero for less than one month<br>I don't know<br>No answer	[__I__]<br>[__I__]<br>0<br>888<br>999	
**Q204**	Currently do live with your husband or alone?	Married and living with the spouse<br>Married but living with other partners<br>Married and living alone<br>Common-law relationship<br>Not married and alone<br>I don't know<br>No answer	1<br>2<br>3<br>4<br>5<br>888<br>999	
**Q205**	Do you suspect that your husband could have extra marital sexual relations during the last 6 months?	Yes<br>No<br>I don't know<br>No answer	1<br>2<br>888<br>999	
**Q206**	Does your husband have other women or sexual partners, publicly or in secret?	Number of partners<br>I don't know<br>No answer	[__I__]<br>888<br>999	

Recruitment form (16-20 WG)

Q207	How many sexual partners did you have during the last six months?	Number of sexual partners<br>I don't know<br>No answer	[__I__]<br>888<br>999	
Q208	How many sexual partners did you have within the lifetime?	Number of sexual partners 1<br>2<br>3<br>4<br>More than 4<br>I don't know<br>No answer	1<br>2<br>3<br>4<br>5<br>888<br>999	
Q209	What is your income source?<br><br>NB: several answers are possible	Street vendor<br>Housework with economic activity<br>Housekeeper<br>Local beverage vendor<br>dressmaker<br>Farmer<br>Secretary<br>Haiddresser<br>Teacher<br>Market porter<br>Fresh provisions vendor<br>Housewife without econimic activity<br>Other……………………………………<br>I don't know<br>No answer	1<br>2<br>3<br>4<br>5<br>6<br>7<br>8<br>9<br>10<br>11<br>12<br>13<br>888<br>999	
Q210	What is the age at first sex?	years<br>I don't know<br>No answer	[__I__]<br>888<br>999	
Q211	Have you ever had anal sex?	Yes<br>No<br>I don't know<br>No answer	1<br>2<br>888<br>999	

N°	Filter questions	Coding		
**Section 3 : Telephone use. Now I will ask you the questions about how you use the mobile phone**				
Q301	What networks do you use to communicate?<br>(multiple answer)	Airtel<br>orange<br>Vodacom<br>MTN<br>Tigo<br>None of those mentioned before<br>no phone<br>I don't know<br>No answer	1<br>2<br>3<br>4<br>5<br>6<br>7<br>888<br>999	
Q302	What type of communication do you use?<br>(multiple answer)	SMS<br>Call<br>Social media<br>Internet<br>Other<br>I don't know<br>No answer	1<br>2<br>3<br>4<br>5<br>888<br>999	
Q303	When is the best time to communicate?<br><br>(multiple answer)	morning<br>Miidday<br>Evening<br>Other<br>I don't know	1<br>2<br>3<br>4<br>888	

Recruitment form (16-20 WG)

		Pas de réponse	999	
Q304	Weekly average of top up phone credit	Less than one dollar<br>1 dollar<br>between1 and 5 dollars<br>between 5 et 10 dollars<br>more than 10 dollars<br>Other: _______________<br>I don't know<br>No answer	1<br>2<br>3<br>4<br>5<br>6<br>888<br>999	
Q305	Have you ever used the phone to contact health care staff?	Yes<br>No<br>I don't know<br>No answer	1<br>2<br>888<br>999	
Q306	What prompted you to contact him?	Advice<br>Appointment<br>Emergency<br>Other: ______________________<br>I don't know<br>No answer	1<br>2<br>3<br>4<br>888<br>999	
Q307	Would you like to contact an health care staff?	Yes<br>No<br>I don't know<br>No answer	1<br>2<br>888<br>999	If answer other than 1 to this question move to Q309
Q308	If yes, why?	Health advice<br>Reminder of an appointment<br>Dosage of a drug<br>Lab result<br>Other: _________________<br>I don't know<br>No answer	1<br>2<br>3<br>4<br>5<br>888<br>999	
Q309	Does your husband or partner have a phone? can we use it to contact you?	Yes<br>No<br>I don't know<br>No answer	1<br>2<br>888<br>999	
Q310	In your entourage or neighborhood is there a telephone that can be used to contact you?	yes<br>No<br>I don't know<br>No answer	1<br>2<br>888<br>999	If answer other than 1 to this question move to Q312
Q311	If so, who has a phone that can be used to contact you?	Close neighbor<br>Family member living in the same house<br>Neighborhood chief<br>Payphone in the neighborhood<br>Other: _________________________<br>I don't know<br>No answer	1<br>2<br>3<br>4<br>5<br>888<br>999	
Q312	How do you charge your phone battery?	Electricity power<br>Generator<br>Solar energy<br>Manual recharge<br>Other:_______________________<br>I don't know<br>No answer	1<br>2<br>3<br>4<br>5<br>888<br>999	

Recruitment form (16-20 WG) R

N°	Filter questions	Coding		
**Section 4: Personal hygiene. Now, we will ask questions about the hygiene of intimate areas**				
**Q401**	How do you do your vaginal hygiene?	External hygiene<br>External application of product<br>Washing by water<br>Washing by soap<br>Use fragrance<br>Use powder<br>Use of water mixed with lemon juice<br>Use of Dettol/Germol<br>Use of the soap virginity<br>Other : ______________________<br>I don't know<br>No answer	1<br>2<br>3<br>4<br>5<br>6<br>7<br>8<br>9<br>10<br>888<br>999	
**Q402**	Do you use practices to make the vagina dry, warmer, tighter or to modify the smell?	Yes<br>No<br>I don't know<br>No answer	1<br>2<br>888<br>999	If answer other than 1 to this question move to Q404
**Q403**	What are practices used for this purpose?	External hygiene<br>External application of product<br>Washing by water<br>Washing by soap<br>Use fragrance<br>Use powder<br>Use of water mixed with lemon juice<br>Use of Dettol/Germol<br>Use of the soap virginity<br>Shaving<br>Other: ______________________<br>I don't know<br>No answer	1<br>2<br>3<br>4<br>5<br>6<br>7<br>8<br>9<br>10<br>11<br>888<br>999	
**Q404**	How often do you do your personal hygiene?	More than twice per day<br>Twice per day<br>Once per day<br>Once per week<br>Once per month<br>Other……………………..<br>I don't know<br>No answer	1<br>2<br>3<br>4<br>5<br>6<br>888<br>999	
**Q405**	Do you clean yourself after each sexual intercourse (personal hygiene)?	Yes<br>No<br>I don't know<br>No answer	1<br>2<br>888<br>999	
**Q406**	What do you use for this personal toilet?	Water<br>wipes<br>Other :_____________	1<br>2<br>3	
**Q407**	Is your husband or partner circumcised?	Yes<br>No<br>I don't know<br>No answer	1<br>2<br>888<br>999	

R

Recruitment form (16-20 WG)

**Q408**	In some communities there is the practice of labia elongation. Did you do it?	Yes<br>No<br>I don't know<br>No answer	1<br>2<br>888<br>999	

**N°**	**Filter questions**	**Coding**		
**Section 5 : Alcohol, food, autres substances. Now we are going to ask questions about some eating habits and lifestyle**				
**Q501**	Do you drink alcohol? By alcohol we mean local drinks (Kasisksi,musululu,mandrakua,..), beer,wine or liqueur	Yes<br>No<br>I don't know<br>No answer	1<br>2<br>888<br>999	If answer other than 1 to this question move to Q503
**Q502**	What kind of alcohol do you drink?	Beer<br>Wine<br>Liqueur<br>Local drinks (made from maize, banana or sorghum)<br>Other<br>I don't know<br>No answer	1<br>2<br>3<br>4<br>5<br>888<br>999	
**Q503**	When was the last time you drank alcohol?	Today<br>Yesterday<br>A week ago<br>Two weeks ago<br>One month ago<br>Three months ago<br>I don't know<br>No answer	1<br>2<br>3<br>4<br>5<br>6<br>888<br>999	
**Q504**	If so, what quantity?	Once per day<br>More than once per day<br>Once per week<br>Several times a week: _______/sem<br>I don't know<br>No answer	1<br>2<br>3<br>4<br>888<br>999	
**Q505**	Do you ever eat clay (mabele)?	Yes<br>No<br>I don't know<br>No answer	1<br>2<br>888<br>999	If answer other than 1 to this question move to Q506
**Q506**	Do you ever consume charcoal?	Yes<br>No<br>I don't know<br>No answer	1<br>2<br>888<br>999	
**Q507**	For how long are you consuming clay or charcoal?	One week<br>Two weeks<br>One month<br>More than three months<br>More than six months<br>Others:_________________	1<br>2<br>3<br>4<br>5<br>6	
**Q508**	Do you ever use tobacco?	Yes<br>No	1<br>2	
**Q509**	Do you ever use natural stimulants or drug (kat,ginger,hemp).	Yes<br>No	1<br>2	

**N°**	**Filter questions**	**Coding**

R

Recruitment form (16-20 WG)

Section 6: Genital infections				
**Q601**	During antenatal consultation,HIV is checked.<br>Do you know your HiV status?	Yes<br>No<br>I don't know<br>No answer	1<br>2<br>888<br>999	If answer other than 1 to this question move to Q603
**Q602**	When was the last time you had the HIV test?	< 6 months<br>6 months – year<br>> one year<br>I don't know<br>No answer	1<br>2<br>3<br>888<br>999	
**Q603**	Do you know the serological HIV status of your husband?	Yes<br>No<br>I don't know<br>No answer	1<br>2<br>888<br>999	
**Q604**	Have you ever had the couple's HIV test?	Yes<br>No<br>I don't know<br>No answer	1<br>2<br>888<br>999	
**Q605**	Have you had any vaginal discharge during pregnancy?	Yes<br>No<br>I don't know<br>No answer	1<br>2<br>888<br>999	If answer other than 1 to this question move to Q608
**Q606**	Have you received treatment?	Yes<br>No<br>I dont know<br>No answer	1<br>2<br>888<br>999	
**Q607**	If yes, what medication?	Name /PO/IM/IV<br>vaginal/dermal	______	
**Q608**	Do you have vaginal itching during this pregnancy?	Yes<br>No<br>I don't know<br>No answer	1<br>2<br>888<br>999	If answer other than 1 to this question move to Q611
**Q609**	Have you received treatment?	Yes<br>No<br>I don't know<br>No answer	1<br>2<br>888<br>999	
**Q610**	If yes, what medication?	No /PO/IM/IV<br>vaginal/dermal	______	
**Q611**	Have you had mictalgia during pregnancy?	Yes<br>No<br>I don't know<br>No answer	1<br>2<br>888<br>999	If answer other than 1 to this question move to Q614
**Q612**	Have you received treatment?	Yes<br>No<br>I don't know<br>No answer	1<br>2<br>888<br>999	
**Q613**	If yes, what medication?	Name /PO/IM/IV/<br>vaginal/dermal	______	
**Q614**	Have you had vaginal burning sensation after sex?	Yes<br>No<br>I don't know<br>No answer	1<br>2<br>888<br>999	If answer other than 1 to this question move to Q618

Recruitment form (16-20 WG)

**Q615**	The last time you had this burning sensation	Today<br>2 days ago<br>2-7 days ago<br>7-14 days ago<br>< 14 days ago<br>I don't know<br>No answer	1<br>2<br>3<br>4<br>5<br>888<br>999	
**Q616**	Have you received treatment?	Yes<br>No<br>I don't know<br>No answer	1<br>2<br>888<br>999	
**Q617**	If yes, what medication?	Name /PO/IM/IV<br>vaginal/dermal		
**Q618**	Have you had a feeling of vagina odor during this pregnancy?	Yes<br>No<br>I don't know<br>No answer	1<br>2<br>888<br>999	If answer other than 1 to this question move to Q622
**Q619**	The last time you had this bad smell.	Today<br>Two days ago<br>2-7 days ago<br>7-14days ago<br>>14 days ago<br>I don't know<br>No answer	1<br>2<br>3<br>4<br>5<br>888<br>999	
**Q620**	Have you received treatment?	Yes<br>No<br>I don't know<br>No answer	1<br>2<br>888<br>999	
**Q621**	If yes, what medication?	Nom /PO/IM/IVva<br>ginal/dermique		
**Q622**	Have you received any medicine of Gonorrhea or Syphilis?	Yes<br>No<br>I don't know<br>No answer	1<br>2<br>888<br>999	
**Q623**	If yes, what medication?	Name /PO/IM/IV<br>vaginal/dermal		
**Q624**	Are you currently taking any medication? By drug we mean that prescribed by a Doctor, Nurse, Pharmacist or that you buy yourself.	Yes<br>No<br>I don't know<br>No answer	1<br>2<br>888<br>999	If answer other than 1 to this question move to Q626
**Q625**	If yes, what medication?	Name /PO/IM/IV<br>vaginal/dermal		
**Q626**	Are you diabetic?	Yes<br>No<br>I don't know<br>No answer	1<br>2<br>888<br>999	
**Q627**	Are there diabetics in your family?	Yes<br>No<br>I don't know<br>No answer	1<br>2<br>888<br>999	

Recruitment form (16-20 WG)

**Q628**	Do you suffer from a chronic disease other than diabetes?	Yes<br>No<br>I don't know<br>No answer	1<br>2<br>888<br>999	

**N°**	**Filter questions**	**Coding**		
**Section 7 : Obstetric history**				
**Q701**	How many full term deliveries?	Number	[__I__]	
**Q702**	How many premature births?	Number	[__I__]	
**Q703**	How many abortions?	Number	[__I__]	
**Q704**	How many fetal death?	Number	[__I__]	
**Q705**	The biggest baby weight?	Weight in gram	[__I__][__I__]	
**Q706**	How many cesarean births	Never<br>1<br>2<br>3<br>>3<br>I don't know<br>No answer	1<br>2<br>3<br>4<br>5<br>888<br>999	
**Q707**	Have you ever had a newborn with neonatal infection?	Yes<br>No<br>I don't know<br>No answer	1<br>2<br>888<br>999	If answer other than 1 to this question move to Q709
**Q708**	What was the course of the disease?	Discharge<br>Death<br>Disability	1<br>2<br>3	
**Q709**	Do you use mosquito net?	Yes<br>No<br>I don't know<br>No answer	1<br>2<br>888<br>999	
**Q710**	Do you use malaria prophylaxis?	Yes<br>No<br>I don't know<br>No answer	1<br>2<br>888<br>999	
**Q711**	Do you use worms prophylaxis?	Yes<br>No<br>I don't know<br>No answer	1<br>2<br>888<br>999	
**Q712**	How many antenatal care consultations have you done?	First time<br>second time<br>Third time<br>Other:_________<br>I don't know<br>No answer	1<br>2<br>3<br>4<br>888<br>999	

Recruitment form (16-20 WG)

**Q713**	Do you have constipation?	Yes<br>No<br>I don't know<br>No answer	1<br>2<br>888<br>999	If answer other than 1 to this question move to Q709
**Q714**	Do you have enemas to heal constipation?	Yes<br>No<br>I don't know<br>No answer	1<br>2<br>888<br>999	
**Q715**	Do you have cold sore rashes on the vulva?	Yes<br>No<br>I don't know<br>No answer	1<br>2<br>888<br>999	
**Q716**	Do you use substances (plants, leaves, seeds, etc.) to fight against neonatal infection?	Yes<br>No<br>I don't know<br>No answer	1<br>2<br>888<br>999	
**Q717**	If yes, which ones do you use?	name	______	
**Section 8: Current Pregnancy. Now we are going to ask about the pregnancy you are carrying?**				
**Q801**	When was the last sexual intercourse?	Today<br>yesterday<br>less than 7 days<br>less than one month<br>Between 1-3 months<br>More than 3months<br>I don't know<br>No answer	1<br>2<br>3<br>4<br>5<br>6<br>888<br>999	
**Q802**	Do you know your weight before conception?	Weight in Kgs	[__I__][__I	
**Q803**	Do you have fever?	Yes<br>No<br>I don't know<br>No answer	1<br>2<br>888<br>999	
**Q804**	Do you have headache?	Yes<br>No<br>I don't know<br>No answer	1<br>2<br>888<br>999	
**Q805**	Do you have cough?	Yes<br>No<br>I don't know<br>No answer	1<br>2<br>888<br>999	
**Q806**	Do you have uterine contractions?	Yes<br>No<br>I don't know<br>No answer	1<br>2<br>888<br>999	
**Q807**	Do you have back pain?	Yes<br>No<br>I don't know<br>No answer	1<br>2<br>888<br>999	
**Q809**	Do you have trouble to swallow?	Yes<br>No<br>I don't know<br>No answer	1<br>2<br>888<br>999	

Recruitment form (16-20 WG)

**Q810**	Have you taken antibiotics in the past two weeks?	Yes<br>No<br>I don't know<br>No answer	1<br>2<br>888<br>999	
**Q811**	If yes, what antibiotic?	Name	______	
**N°**	**Filter questions**	**coding**		
**Section 9: Anthropometrics parameters**				
**Q901**	Current weight	kgs	[__I__][__]	
**Q902**	Height	cm	[__I__][__]	
**Q903**	Midd-Upper Arm Circumference	MUAC cm	[__I__][__]	
**Q904**	Blood pressure	mmHg	[__I__][__][/__I__][__]	
**Q905**	Cardiac frequency	bpm	[__I__][__]	
**Q906**	Oedema of the lower limbs	Yes<br>No	1<br>2	
**Section 10: Clinical exam. Now, we are going to do the clinical examination and take different samples, it want hurt you and will take few minutes**				
**Q1001**	General state	Good<br>worse	1<br>2	
**Q1002**	Vulva	Normal<br>Condyloma<br>Herpetic lesions<br>Chancre<br>Erythema<br>Pustules<br>Bartholin gland abscess<br>Other: ____________________	1<br>2<br>3<br>4<br>5<br>6<br>7<br>8	
**Q1003**	Speculum	Normal<br>Redness<br>Polyp<br>Ectropion<br>Ex utero bleeding<br>Plaques « yellowish sand »<br>Ulcerations<br>Other: ____________________	1<br>2<br>3<br>4<br>5<br>6<br>7<br>8	
**Q1004**	PH	Number	[__I__]	
**Q1005**	Vaginal secretions	Fine texture and homogeneous<br>Thick<br>Thick and heterogeneous	1<br>2<br>3	

**N°**	**Filter questions**	**Coding**		
**Section 11: Ultrasound**				
**Q1101**	GA :(in weeks)	Week of gestation	I__][__][/__I__][__]	
**Q1102**	Estimated fetal weight	grams	[__][/__I__][__]	
**Q1103**	Estimated fetal weight by percentile	Percentile	I__][__]	
**Q1103**	Gender:	Male<br>Female	1<br>2	
**Q1104**	Placenta location	Normal<br>Low-lying placenta	1<br>2	
**Q1106**	Amniotic liquid	AFI : [cm	/__I__]	

Recruitment form (16-20 WG)

**Q1107**	Cervix length	in cm	[/__I__][__]	
**Q1108**	Funnel	Yes<br>No	1<br>2	
**Q1109**	visible deformities	Yes<br>No	1<br>2	

Lab results

N°	Filter questions	Coding		
**Q1201**	wet mount	WBC/field:<br>clue cells number:<br>Trichomonas:<br>Yeast:<br>epithelial cells<br><br>Other :________________	[__I__]<br>[__I__]<br>[__I__]<br>[__I__]<br>[__I__]<br>[__I__]	
**Q1202**	Whiff test (KOH) test	Positive<br>Negative	1<br>2	
**Q1203**	Score AV of donders	<3 no sign of AV<br>3-4 Light AV<br>5-6 Moderate AV<br>> 6 Severe AV	1<br>2<br>3<br>4	
**Q1204**	Amsel criteria	Number		
**Q1205**	Hemoglobin rate (hemocue test)	g/dl		
**Q1206**	Rapid malaria test	Positive<br>Negative	1<br>2	
**Q1207**	HIV rapid test	Postive<br>Negative	1<br>2	
**Q1208**	Urine dipstick(nitrite)	Positive<br>Negative	1<br>2	
**Q1209**	Urine dipstick(leukocyturia)	Positive<br>Negative	1<br>2	
**Q12010**	Glycated keratin	Normal<br>Anormal	1<br>2	
**Q12011**	Diagnosis	normal<br>Abnormal	1<br>2	
**Q12013**	Condition	name		
**Q1017**	Provided treatment	Medicine	________________	

Confirmation of sample collection Yes ☐ No☐
Validated by Dr ……………………………… Date ……. /…………./………………
Signature
