# Supplementary material for: Prevalence, risk factors and adverse pregnancy outcomes of second trimester bacterial vaginosis among pregnant women in Bukavu, Democratic Republic of the Congo
Source: PLoS One. 2021 Oct 25;16(10):e0257939. doi: 10.1371/journal.pone.0257939 (PMC8544863; doi:10.1371/journal.pone.0257939)
Supplement: S2 File — (DOCX) [file pone.0257939.s002.docx]

**Supplementary Information 2**

**Table 1. Pregnant women’s sociodemographic, anthropometric, sexual and hygiene behaviors and Clinical and laboratory findings stratified by second trimester Nugent score results (525)**

| **Characteristics of pregnant women** | **Number**  **525* (100)** | **Nugent score patterns** | | |
| --- | --- | --- | --- | --- |
|  |  | **Healthy**  **285 (54.3)** | **Intermediate flora**  **102 (19.4)** | **BV**  **138 (26.3)** |
| ***Sociodemographic characteristics*** |  |  |  |  |
| **Age at recruitment (years)** |  |  |  |  |
| <20 years | 26 (5.0) | 13 (4.6) | 5 (4.9) | 8 (5.8) |
| 20-24 years | 113 (21.5) | 65 (22.8) | 17 (16.7) | 31 (22.5) |
| 25-29 years | 173 (33.0) | 94 (33.0) | 36 (35.3) | 43 (31.2) |
| 30-34 years | 135 (25.7) | 72 (25.3) | 30 (29.4) | 33 (23.9) |
| ≥35 years | 78 (14.9) | 41 (14.4) | 14 (13.7) | 23 (16.7) |
| **Tribe ^(1)^** |  |  |  |  |
| Shi | 374 (71.2) | 203 (71.2) | 83 (81.4) | 88 (63.8) |
| Rega | 57 (10.9) | 32 (11.2) | 8 (7.8) | 17 (12.3) |
| Other tribes | 94 (17.9) | 50 (17.5) | 11 (10.8) | 33 (23.9) |
| **Religion ^(2)^** |  |  |  |  |
| Christians | 492 (93.7) | 266 (93.3) | 94 (92.2) | 132 (95.7) |
| Not Christian | 33 (6.3) | 19 (6.7) | 8 (7.8) | 6 (4.4) |
| **Education level ^(3)^** |  |  |  |  |
| Primary | 62 (11.8) | 35 (12.3) | 11 (10.8) | 16 (11.6) |
| Secondary | 273 (52.0) | 151 (53.0) | 54 (52.9) | 68 (49.3) |
| Higher | 190 (36.2) | 99 (34.7) | 37 (36.3) | 54 (39.1) |
| **Quality of life ^(4)^** |  |  |  |  |
| Poor | 381 (72.6) | 209 (73.3) | 74 (72.6) | 98 (71.0) |
| No poor | 144 (27.4) | 76 (26.7) | 28 (27.5) | 40 (29.0) |
| **Employment status** |  |  |  |  |
| Employed or self employed | 89 (17.0) | 57 (20.0) | 17 (16.7) | 15 (10.9) |
| Unemployed | 436 (83.1) | 228 (80.0) | 85 (83.3) | 123 (89.1) |
| **Marital status** |  |  |  |  |
| Married | 501 (95.4) | 273 (95.8) | 100 (98.0) | 128 (92.8) |
| Not married | 24 (4.6) | 12 (4.2) | 2 (2.0) | 10 (7.3) |
| **Alcohol consumption ^(5)^** |  |  |  |  |
| Yes | 190 (36.2) | 91 (31.9) | 45 (44.1) | 54 (39.1) |
| No | 335 (63.8) | 194 (68.1) | 57 (55.9) | 84 (60.9) |
| **Clay consumption ^(6)^** |  |  |  |  |
| Yes | 141 (26.9) | 103 (36.1) | 11 (10.8) | 27 (19.6) |
| No | 384 (73.1) | 182 (63.9) | 91 (89.2) | 111 (80.4) |
| **Tobacco use ^(7)^** |  |  |  |  |
| Yes | 2(0.4) | 0 (0.00) | 0 (0.00) | 2 (1.5) |
| ***Sexual behaviour characteristics*** |  |  |  |  |
| **Age at marriage** |  |  |  |  |
| ≤ 18 years | 118 (22.5) | 64 (22.5) | 26 (25.5) | 28 (20.3) |
| > 18 years | 407 (77.5) | 221 (77.5) | 76 (74.5) | 110 (79.7) |
| **Duration of marriage** |  |  |  |  |
| ≤ 5 years | 288 (54.9) | 160 (56.1) | 56 (54.9) | 72 (52.2) |
| >5 years | 237 (45.1) | 125 (43.9) | 46 (45.1) | 66 (47.8) |
| **Husband has had concurrent extra-marital female sexual partners in the last six months** ^(8)^ |  |  |  |  |
| Yes | 51 (9.7) | 22 (7.7) | 7 (6.9) | 22 (15.9) |
| No | 474 (90.3) | 263 (92.3) | 95 (93.1) | 116 (84.1) |
| **Pregnant woman has had concurrent extra-marital male sexual partners in the last six months** ^(9)^ |  |  |  |  |
| No | 515 (98.1) | 282 (99.0) | 101 (99.0) | 132 (95.7) |
| Yes | 10 (1.9) | 3 (1.1) | 1 (1.0) | 6 (4.4) |
| **Partners during lifetime** |  |  |  |  |
| One | 292 (55.6) | 160 (56.1) | 65 (63.7) | 67 (48.6) |
| More than one | 233 (44.4) | 125 (43.9) | 37 (36.3) | 71 (51.5) |
| **Age of first sexual intercourse** |  |  |  |  |
| <18 | 156 (29.7) | 76 (26.7) | 32 (31.4) | 48 (34.8) |
| ≥18 | 369 (70.3) | 209 (73.3) | 70 (68.6) | 90 (65.2) |
| **Anal intercourse practice** |  |  |  |  |
| Yes | 52 (9.9) | 32 (11.2) | 5 (4.9) | 15 (10.9) |
| No | 473 (90.1) | 253 (88.8) | 97 (95.1) | 123 (89.1) |
| **Husband circumcision** |  |  |  |  |
| Circumcised | 507 (96.6) | 276 (96.8) | 99 (97.1) | 132 (95.7) |
| No circumcised | 18 (3.4) | 9 (3.2) | 3 (2.9) | 6 (4.4) |
| **Lengthening of lips ^(10)^** |  |  |  |  |
| Yes | 63 (12.0) | 31 (10.9) | 12 (11.8) | 20 (14.5) |
| No | 462 (88.0) | 254 (89.1) | 90 (88.2) | 118 (85.5) |
| ***Sanitation and hygiene characteristics*** |  |  |  |  |
| **Types of toilet** |  |  |  |  |
| Flushing toilet | 224 (42.7) | 122 (42.8) | 39 (38.2) | 63 (45.7) |
| Pit toilet | 301 (57.3) | 163 (57.2) | 63 (61.8) | 75 (54.4) |
| **Mode of cleaning after toilet** |  |  |  |  |
| Water | 351 (66.9) | 183 (64.2) | 73 (71.6) | 95 (68.8) |
| Toilet paper/ wet wipes | 174 (33.1) | 102 (35.8) | 29 (28.4) | 43 (31.2) |
| **Substances used during intimate toilet ^(11)^** |  |  |  |  |
| water only | 426 (81.1) | 237 (83.2) | 89 (87.3) | 100 (72.5) |
| Varied substances | 99 (18.9) | 48 (16.8) | 13 (12.8) | 38 (27.5) |
| **Number of intimate toilets per day ^(12)^** |  |  |  |  |
| ≤2 per day | 292 (55.6) | 177 (62.1) | 45 (44.1) | 70 (50.7) |
| >2 per day | 233 (44.4) | 108 (37.9) | 57 (55.9) | 68 (49.3) |
| ***Obstetrical characteristics*** |  |  |  |  |
| **Parity** |  |  |  |  |
| 0 | 137 (26.1) | 81 (28.4) | 23 (22.6) | 33 (23.9) |
| 1 | 81 (15.4) | 47 (16.5) | 11 (10.8) | 23 (16.7) |
| 2 | 89 (17.0) | 42 (14.7) | 23 (22.6) | 24 (17.4) |
| ≥3 | 218 (41.5) | 115 (40.4) | 45 (44.1) | 58 (42.0) |
| **Previous PTB** |  |  |  |  |
| No | 500 (95.2) | 273 (95.8) | 94 (92.2) | 133 (96.4) |
| Yes | 25 (4.8) | 12 (4.2) | 8 (7.8) | 5 (3.6) |
| **History of vaginal infection ^(13)^** |  |  |  |  |
| Yes | 12 (2.3) | 7 (2.5) | 2 (2.0) | 3 (2.2) |
| No | 513 (97.7) | 278 (97.5) | 100 (98.0) | 135 (97.8) |
| **Use of mosquito net** |  |  |  |  |
| Yes | 449 (85.5) | 243 (85.3) | 88 (86.3) | 118 (85.5) |
| No | 76 (14.5) | 42 (14.7) | 14 (13.7) | 20 (14.5) |
| **History of a consultation during this pregnancy** |  |  |  |  |
| Not yet | 505 (96.2) | 272 (95.4) | 100 (98.0) | 133 (96.4) |
| Yes | 20 (3.8) | 13 (4.6) | 2 (2.0) | 5 (3.6) |
| ***Clinical, and anthropometrics findings*** |  |  |  |  |
| **Clinical status ^(14)^ at first visit** |  |  |  |  |
| Symptomatic | 254 (48.4) | 126 (44.2) | 55 (53.9) | 73 (52.9) |
| Asymptomatic | 271 (51.6) | 159 (55.8) | 47 (46.1) | 65 (47.1) |
| **Current abnormal discharge** |  |  |  |  |
| Yes | 251 (47.8) | 115 (40.4) | 63 (61.8) | 73 (52.9) |
| No | 274 (52.2) | 170 (59.7) | 39 (38.2) | 65 (47.1) |
| **Current vaginal itching** |  |  |  |  |
| Yes | 215 (41.0) | 96 (33.7) | 51 (50.0) | 68 (49.3) |
| No | 310 (59.1) | 189 (66.3) | 51 (50.0) | 70 (50.7) |
| **Current dysuria** |  |  |  |  |
| Yes | 140 (26.8) | 74 (26.0) | 25 (24.8) | 41 (29.9) |
| No | 383 (73.2) | 211 (74.0) | 76 (75.3) | 96 (70.1) |
| **Burning sensation after Sexual intercourse** |  |  |  |  |
| Yes | 171 (32.6) | 88 (30.9) | 34 (33.3) | 49 (35.5) |
| No | 354 (67.4) | 197 (69.1) | 68 (66.7) | 89 (64.5) |
| **Current abnormal vaginal foul smell** |  |  |  |  |
| Yes | 114 (21.7) | 56 (19.7) | 23 (22.6) | 35 (25.4) |
| No | 411 (78.3) | 229 (80.4) | 79 (77.5) | 103 (74.6) |
| **BMI ^(15)^** |  |  |  |  |
| Lean (<18.5–24.9 kg/m^2^) | 219 (41.7) | 119 (41.8) | 42 (41.2) | 58 (42.0) |
| Overweight (≥25.0–29.9 kg/m^2^) | 199 (37.9) | 114 (40.0) | 30 (29.4) | 55 (39.9) |
| Class I obese (30.0– 34.9 kg/m^2^ ) | 83 (15.8) | 43 (15.1) | 22 (21.6) | 18 (13.0) |
| Class II/III obese (35.0– ≥40.0 kg/m^2^) | 24 (4.6) | 9 (3.2) | 8 (7.8) | 7 (5.1) |
| **Cervix length** |  |  |  |  |
| <30 mm | 52 (9.9) | 31 (10.9) | 6 (5.9) | 15 (10.9) |
| ≥30mm | 473 (90.1) | 254 (89.1) | 96 (94.1) | 123 (89.1) |
| **MUAC at first visit ^(16)^** |  |  |  |  |
| <22cm | 51 (9.7) | 28 (9.8) | 13 (12.8) | 10 (7.3) |
| ≥22cm | 474 (90.3) | 257 (90.2) | 89 (87.3) | 128 (92.8) |
| ***Laboratory findings*** |  |  |  |  |
| **Vaginal pH** |  |  |  |  |
| ≤4.5 | 8 (1.5) | 7 (2.5) | 1 (1.0) | 0 (0.00) |
| 4.6–6 | 406 (77.3) | 232 (81.4) | 78 (76.5) | 96 (69.6) |
| >6 | 111 (21.1) | 46 (16.1) | 23 (22.6) | 42 (30.4) |
| **Whiff test** |  |  |  |  |
| Positive | 47 (9.0) | 11 (3.9) | 9 (8.8) | 27 (19.6) |
| Negative | 478 (91.1) | 274 (96.1) | 93 (91.2) | 111 (80.4) |
| **Presence of Clue cells on wet mount** |  |  |  |  |
| Yes | 55 (10.5) | 40 (14.0) | 5 (4.9) | 10 (7.3) |
| No | 470 (89.5) | 245 (86.0) | 97 (95.1) | 128 (92.8) |
| **Amsel score** |  |  |  |  |
| Abnormal | 40 (7.6) | 15 (5.3) | 7 (6.9) | 18 (13.0) |
| Normal | 485 (92.4) | 270 (94.7) | 95 (93.1) | 120 (87.0) |
| **Adjusted Haemoglobin ^(17)^** |  |  |  |  |
| <110 g/L | 117 (22.3) | 65 (22.8) | 21 (20.6) | 31 (22.5) |
| ≥110.1g/L | 408 (77.7) | 220 (77.2) | 81 (79.4) | 107 (77.5) |
| **Malaria rapid test ^(18)^** |  |  |  |  |
| Positive | 2 (0.4) | 1 (0.4) | 1 (1.0) | 0 (0.00) |
| Negative | 523 (99.6) | 284 (99.7) | 101 (99.0) | 138 (100) |
| **Rapid HIV test ^(19)^** |  |  |  |  |
| Positive | 2 (0.4) | 0 (0.0) | 0 (0.00) | 2 (1.5) |
| Negative | 523 (99.6) | 285 (100.0) | 102 (100.0) | 136 (98.6) |
| **Trichomonas on Wet Mount** |  |  |  |  |
| Yes | 7 (1.3) | 3 (1.1) | 2 (2.0) | 2 (1.5) |
| No | 518 (98.7) | 282 (99.0) | 100 (98.0) | 136 (98.6) |
| **Candida on wet mount** |  |  |  |  |
| Yes | 138 (26.3) | 63 (22.1) | 34 (33.3) | 41 (29.7) |
| No | 387 (73.7) | 222 (77.9) | 68 (66.7) | 97 (70.3) |
| **Candida on Gram stain** |  |  |  |  |
| Yes | 148 (28.2) | 57 (20.0) | 42 (41.2) | 49 (35.5) |
| No | 377 (71.8) | 228 (80.0) | 60 (58.8) | 89 (64.5) |
| **Biofilm on gram stain** |  |  |  |  |
| Yes | 110 (21.1) | 7 (2.5) | 24 (23.5) | 80 (58.0) |
| No | 414 (78.9) | 278 (97.5) | 78 (76.5) | 58 (42.0) |

* Eight slides did not contain biological material or appeared damaged.

1. Other tribes (Tembo, Fuliru, Hunde, Nyanga, Hutu, Nande, Vira, Bembe) proportion for each tribe was less than 2.5%
2. The sub heading Christian represents Catholics, protestants, Anglicans, Kimbanguistes and members of revival church participants. And the sub heading not Christian represents Muslim, Atheist, Animist, and nonbelievers’ participants
3. Each of three maternal education levels contains participants who completely end the level and the ones who not completely end the degree.
4. Taking into account local parameters, poverty was calculated considering the type of the floor, water source, electricity, commodities in the house; the total score was ranging from 4 to 17. Pregnant women who got a score less than 10 were considered like living under the threshold of poverty and the ones with a score ≥10 were considered living above the threshold of poverty. We did not include the income of participant because it is very instable and depends mainly from the informal sector.
5. Alcohol consumption**:** more or equal to one or two glasses of 30 cl per day of local beer ,4–5.5% Alcohol content
6. Clay consumption: more or equal to a regular basis consumption during current pregnancy (approximately 20 gr per take 5 time (100 mg) per day)
7. Tobacco use: use of cigarette, chew tobacco, cigar, smoking pipe
8. Husband has had concurrent extra-marital female sexual partners in the last six months.

Concurrent husband’ partners**:** Known female sexual partnerships of the husband of the pregnant women that overlap in time as opposed to running sequentially (Kenyon 2014)

1. Pregnant woman has had concurrent extra-marital male sexual partners in the last six months.

Concurrent pregnant woman’s partners: male sexual partnerships of the pregnant women in the last six months that overlap in time as opposed to running sequentially (Kenyon 2014)

1. Lengthening lips: an old practice to lengthen outer lips by using herbs during adolescence.
2. Varied substances: soap, herbs, mixed powders, lemon, disinfectant products (chlorhexidine, biseptine, benzalkonium chloride)
3. Intimate toilets: is defined as the process of intravaginal cleansing with liquid solution
4. History of a vaginal infection during the last six months
5. Participants were symptomatic if they presented one or both of these signs abnormal discharge, itching, burning sensation after sexual intercourse, foul smell from vagina
6. BMI: body mass index; lean category was the combination of underweight category with only 8 participants in this study (≤18.5 kg/m^2^ ) and normal range category(18.5–24.9 kg/m^2^ ). Class II/III obese is the combination of severe obesity (35.0–39.9. kg/m^2^) class and very severe obesity (≥40.0 kg/m^2^) class.
7. MUAC: Mid-Upper Arm circumference; measured for early detection of Malnutrition
8. Adjusted hemoglobin: hemoglobin adjusted on the altitude of Bukavu city (1498m) by reducing 20 grams per a liter ^[[1]](#footnote-1)^
9. Rapid test of malaria; SD BIOLINE malaria Ag P.f/Pan is a qualitative and differential test for the detection of histidine-rich proteinII antigen of Plasmodium falciparum and common plasmodium lactate dehydrogenase(pLDH) of plasmodium species in human whole blood.
10. Rapid HIV test; Determine HIV rapid test ALERE DETERMINE HIV ½ used to prevent mother to child transmission.

**Table 2. Adverse pregnancy outcomes stratified by second trimester Nugent score results**

| ***Pregnancy outcomes*** | **330 (100)** | **Healthy** | **Intermediate flora** | **BV** |
| --- | --- | --- | --- | --- |
| **PROM ^(20)^** |  |  |  |  |
| ruptured | 68 (20.6) | 35 (19.2) | 13 (20.3) | 20 (23.8) |
| intact | 262 (79.4) | 147 (80.8) | 51 (79.7) | 64 (76.2) |
| **Apgar at fifth minute** |  |  |  |  |
| ≤7 | 9 (2.7) | 5 (2.8) | 3 (4.8) | 1 (1.2) |
| >7 | 321 (97.3) | 177 (97.3) | 60 (95.2) | 84 (98.8) |
| **Admission to NICU for suspicion of sepsis ^(21)^** |  |  |  |  |
| Yes | 14 (4.2) | 10 (5.5) | 3 (4.7) | 1 (1.2) |
| No | 316 (95.8) | 172 (94.5) | 61 (95.3) | 83 (98.8) |
| **Weight at birth ^(22)^** |  |  |  |  |
| <2500gr | 17 (5.2) | 5 (2.8) | 4 (6.3) | 8 (9.5) |
| ≥2500gr | 313 (94.9) | 177 (97.3) | 60 (93.8) | 76 (90.5) |
| **Gestational age at delivery ^(23)^** |  |  |  |  |
| ≥37weeks GA | 282 (85.5) | 157 (86.3) | 55 (85.9) | 70 (83.3) |
| <37weeksGA | 48 (14.6) | 25 (13.7) | 9 (14.1) | 14 (16.7) |
| **Preterm delivery of low birth weight** | **48 (100)** |  |  |  |
| <2500gr | 15 (31.3) | 4 (16.0) | 4 (44.4) | 7 (50.0) |
| ≥2500gr | 33 (68.8) | 21 (84.0) | 5 (55.6) | 7 (50.0) |

1. Premature rupture of membranes.
2. Early onset neonatal sepsis
3. Low birth weight definition <2500gr^[[2]](#footnote-2)^
4. Gestational age at delivery: the preterm was defined by WHO as delivery at less than 37 completed weeks; less than 259 completed days^[[3]](#footnote-3)^

1. 1. Organization WH. Haemoglobin concentrations for the diagnosis of anaemia and assessment of severity. World Health Organization; 2011. [↑](#footnote-ref-1)
2. 2. Kenyon CR, Osbak K. Recent progress in understanding the epidemiology of bacterial vaginosis. Curr Opin Obstet Gynecol. 2014;26(6):448-54. [↑](#footnote-ref-2)
3. 2. ibid. [↑](#footnote-ref-3)
