# Supplementary material for: Prevalence, risk factors and adverse pregnancy outcomes of second trimester bacterial vaginosis among pregnant women in Bukavu, Democratic Republic of the Congo
Source: PLoS One. 2021 Oct 25;16(10):e0257939. doi: 10.1371/journal.pone.0257939 (PMC8544863; doi:10.1371/journal.pone.0257939)
Supplement: S3 File — (DOCX) [file pone.0257939.s003.docx]

**Supplementary Information 3. Univariate regression analysis of pregnant women’s sociodemographic, anthropometric, sexual and hygiene behaviors and bacterial vaginosis at Visit 1(525*).**

| **Variables** | **N (%)** | **OR (95% CI)** | **p-Value** | |
| --- | --- | --- | --- | --- |
| **Sociodemographic characteristics** |  |  |  | |
| **Age at recruitment (years)** |  |  |  | |
| <20 years | 26 (5.0) | Ref. |  | |
| 20-24 years | 113 (21.5) | 0.85 (0.34–2.16) | 0.733 | |
| 25-29 years | 173 (33.0) | 0.74 (0.30–1.83 | 0.521 | |
| 30-34 years | 135 (25.7) | 0.73 (0.29–1.83) | 0.499 | |
| ≥35 years | 78 (14.9) | 0.94 (0.36–2.47) | 0.902 | |
| **Tribe ^(1)^** |  |  |  | |
| Shi | 374 (71.2) | Ref. |  | |
| Rega | 57 (10.9) | 1.38 (0.75–2.56) | 0.304 | |
| Other tribes | 94 (17.9) | 1.76 (1.08–2.86) | **0.023** | |
| **Religion ^(2)^** |  |  |  | |
| Christian | 492 (93.7) | Ref. |  | |
| Not Christian | 33 (6.3) | 0.61 (0.24–1.50) | 0.279 | |
| **Education ^(3)^** |  |  |  | |
| Higher | 190 (36.2) | Ref. |  | |
| Primary | 62 (11.8) | 0.88 (0.46–1.68) | 0.690 | |
| Secondary | 273 (52.0) | 0.84 (0.55–1.27) | 0.399 | |
| **Quality of life ^(4)^** |  |  |  | |
| No poor | 144 (27.4) | Ref. |  | |
| Poor | 381 (72.6) | 1.11 (0.72–1.71) | 0.633 | |
| **Employment status** |  |  |  | |
| Employed or self employed | 89 (17.0) | Ref. |  | |
| Unemployed | 436 (83.1) | 1.94 (1.07–3.51) | **0.029** | |
| **Marital status** |  |  |  | |
| Married | 501 (95.4) | Ref. |  | |
| Not married | 24 (4.6) | 2.08 (0.90–4.80) | 0.086 | |
| **Alcohol consumption ^(5)^** |  |  |  | |
| No | 335 (63.8) | Ref. |  | |
| Yes | 190 (36.2) | 1.19 (0.79–1.77) | 0.403 | |
| **Clay consumption ^(6)^** |  |  |  | |
| No | 384 (73.1) | Ref. |  | |
| Yes | 141 (26.9) | 0.58 (0.36–0.94) | **0.025** | |
| **Sexual behavior characteristics** |  |  |  | |
| **Age at marriage** |  |  |  | |
| >18 years | 407 (77.5) | Ref. |  | |
| ≤18 years | 118 (22.5) | 0.84 (0.52–1.35) | 0.474 | |
| **Duration of marriage** |  |  |  | |
| > 5 years | 237 (45.1) | Ref. |  | |
| ≤ 5 years | 288 (54.9) | 1.16 (0.78–1.71) | 0.461 | |
| **First sexual intercourse** |  |  |  | |
| ≥18 | 369 (70.3) | Ref. |  | |
| <18 | 156 (29.7) | 1.38 (0.91–2.09) | 0.130 | |
| **Anal intercourse practice** |  |  |  | |
| No | 473 (90.1) | Ref. |  | |
| Yes | 52 (9.9) | 1.15 (0.61–2.17) | 0.659 | |
| **Labia elongation ^(7)^** |  |  |  | |
| No | 462 (88.0) | Ref. |  | |
| Yes | 63 (12.0) | 1.36 (0.77–2.40) | 0.295 | |
| **Pregnant woman has had concurrent extra-marital male sexual partners in the last six months ^(8)^** |  |  |  | |
| No | 515 (98.1) | Ref. |  | |
| Yes | 10 (1.9) | 4.35 (1.21–15.66) | **0.024** | |
| **Circumcision of the husband** |  |  |  | |
| Circumcised | 507 (96.6) |  |  | |
| No circumcised | 18 (3.4) | 1.42 (0.52–3.86) | 0.491 | |
| **Husband has had concurrent extra-marital female sexual partners in the last six months ^(9)^** |  |  |  | |
| No | 474 (90.3) | Ref. |  | |
| Yes | 51 (9.7) | 2.34 (1.29–4.23) | **0.005** | |
| ***Sanitation and hygiene characteristics*** |  |  |  | |
| **Type of toilet** |  |  |  | |
| Flushing toilet | 224 (42.7) | Ref. |  | |
| Pit toilet | 301 (57.3) | 0.85 (0.57–1.25) | 0.409 | |
| **Mode of cleaning after toilet** |  |  |  | |
| Water | 351 (66.9) |  |  | |
| Tissue paper/wet wipes | 174 (33.1) | 0.88 (0.58–1.34) | 0.564 | |
| **Substances used during intimate toilet ^(10)^** |  |  |  | |
| water only | 426 (81.1) |  |  | |
| varied substances | 99 (18.9) | 2.03 (1.28–3.23) | **0.003** | |
| **Number of intimate toilets per day ^(11)^** |  |  |  | |
| ≤ 2 per day | 292 (55.6) | Ref. |  | |
| >2 per day | 233 (44.4) | 1.31 (0.89–1.93) | 0.178 | |
| ***Obstetrical and anthropometrics characteristics*** |  |  |  | |
| **Parity** |  |  |  | |
| ≥3 | 218 (41.5) | Ref. |  | |
| 0 | 137 (26.1) | 0.88 (0.53–1.43) | 0.597 | |
| 1 | 81 (15.4) | 1.09 (0.62–1.93) | 0.757 | |
| 2 | 89 (17.0) | 1.02 (0.58–1.78) | 0.948 | |
| **Previous PTB** |  |  |  | |
| No | 500 (95.2) | Ref. |  | |
| Yes | 25 (4.8) | 0.69 (0.25–1.87) | 0.467 | |
| **History of vaginal infection ^(12)^** |  |  |  | |
| No | 513 (97.7) | Ref. |  | |
| Yes | 12 (2.3) | 0.93 (0.25–3.50) | 0.918 | |
| ***Clinical and laboratory findings*** |  |  |  | |
| **Clinical status at first visit** |  |  |  | |
| Asymptomatic | 271 (51.6) | Ref. |  | |
| Symptomatic | 254 (48.4) | 1.28 (0.87–1.89) | | 0.217 |
| **BMI ^(13)^** |  |  |  | |
| Lean (<18.5–24.9 kg/m^2^) | 219 (41.7) | Ref. |  | |
| Overweight (≥25.0–29.9 kg/m^2^) | 199 (37.9) | 1.06 (0.69–1.63) | 0.791 | |
| class I obese (30.0– 34.9 kg/m^2^) | 83 (15.8) | 0.77 (0.42–1.40) | 0.392 | |
| class II/III obese (35.0– ≥40.0 kg/m^2^) | 24 (4.6) | 1.14 (0.45–2.90) | 0.778 | |
| **MUAC at recruitment ^(14)^** |  |  |  | |
| ≥22cm | 474 (90.3) | Ref. |  | |
| <22cm | 51 (9.7) | 1.52 (0.74–3.12) | 0.257 | |
| **Vaginal *Candida* ^(15)^** |  |  |  | |
| No | 377 (71.8) | Ref. |  | |
| Yes | 148 (28.2) | 1.60 (1.06–2.43) | **0.027** | |
| **Cervix length** |  |  |  | |
| ≥ 30 mm | 473 (90.1) | Ref. |  | |
| <30 mm | 52 (9.9) | 1.15 (0.61–2.17) | 0.659 | |
| **Adjusted maternal haemoglobin^(16)^** |  |  |  | |
| Not anaemic (Hb ≥ 110 g/l) | 408 (77.7) | Ref. |  | |
| Anaemic (Hb < 110 g/l) | 117 (22.3) | 1.01 (0.64–1.62) | 0.953 | |

* Eight slides did not contain biological material or appeared damaged

1. Other tribes (Tembo, Fuliru, Hunde, Nyanga, Hutu, Nande, Vira, Bembe) proportion for each tribe was less than 2.5%
2. The subheading Christian represents Catholics, protestants, Anglicans, Kimbanguistes and members of revival church participants. And the sub heading not Christian represents Muslim, Atheists, Animist, and nonbelievers’ participants
3. Each of three maternal education levels contains participants who completely end the level and the ones who not completely end the degree.
4. Taking into account local parameters, poverty was calculated considering the type of the floor, water source, electricity, commodities in the house; the total score was ranging from 4 to 17. Pregnant women who got a score less than 10 were considered like living under the threshold of poverty and the ones with a score ≥10 were considered living above the threshold of poverty. We did not include the income of participant because it is very instable and depends mainly on the informal sector.
5. Alcohol consumption**:** more or equal to one or two glasses of 30 cl per day of local beer ,4–5.5% Alcohol content
6. Clay consumption: more or equal to a regular basis consumption during current pregnancy (approximately 20 gr per take 5 time per day)
7. Labia elongation: an old practice to lengthen outer lips by using herbs during adolescence.
8. Pregnant woman has had concurrent extra-marital male sexual partners in the last six months. Concurrent pregnant woman’s partners: male sexual partnerships of the pregnant women in the last six months that overlap in time as opposed to running sequentially(1).
9. Husband has had concurrent extra-marital female sexual partners in the last six months. Concurrent husband’ partners**:** Known female sexual partnerships of the husband of the pregnant women that overlap in time as opposed to running sequentially(1).
10. Varied substances: soap, herbs, mixed powders, lemon, disinfectant products (Dettol^R^, benzalkonium chloride,)
11. Intimate toilet is defined as the process of intravaginal cleansing with liquid solution.
12. History of a vaginal infection during the last six months
13. BMI: body mass index; lean category was the combination of underweight category with only 8 participants in this study (≤18.5 kg/m^2^ ) and normal range category(18.5–24.9 kg/m^2^ ). Class II/III obese is the combination of severe obesity (35.0–39.9. kg/m^2^) class and very severe obesity (≥40.0 kg/m^2^) class.
14. MUAC: Mid-Upper Arm circumference; measured for early detection of Malnutrition
15. VVC:  vulvovaginal candidiasis the presence of blastopores, pseudo hyphae and hyphae on Gram stain.
16. Adjusted hemoglobin: hemoglobin adjusted on the altitude of Bukavu city (1498m) by reducing 20 grams per a liter(2).

^[[1]](#footnote-1)^

1. Kenyon CR, Osbak K. Recent progress in understanding the epidemiology of bacterial vaginosis. Curr Opin Obstet Gynecol. 2014;26(6):448-54.

   ^2^ World Health Organization. Haemoglobin concentrations for the diagnosis of anaemia and assessment of severity. World Health Organization; 2011. [↑](#footnote-ref-1)
