# Supplementary material for: Prevalence, risk factors and adverse pregnancy outcomes of second trimester bacterial vaginosis among pregnant women in Bukavu, Democratic Republic of the Congo
Source: PLoS One. 2021 Oct 25;16(10):e0257939. doi: 10.1371/journal.pone.0257939 (PMC8544863; doi:10.1371/journal.pone.0257939)
Supplement: S4 File — (DOCX) [file pone.0257939.s004.docx]

**SI4— Mulinganya et al. STROBE Statement:** Prevalence, risk factors and adverse pregnancy outcomes of bacterial vaginosis among pregnant women in Bukavu, Democratic Republic of the Congo

|  | Item No | Recommendation |
| --- | --- | --- |
| **Title and abstract** | 1 | *(*a) Indicate the study’s design with a commonly used term in the title or the abstract  **Abstract, methods and findings: … pregnant women in the second trimester of pregnancy were recruited and followed until delivery. BV was independently associated with both low birth weight and preterm delivery with associated low birthweight.** |
|  |  | (*b*) Provide in the abstract an informative and balanced summary of what was done and what was found  **A total of 533 pregnant women in the second trimester of pregnancy were recruited and followed until delivery. Clinical and sociodemographic data of mother and newborn and data on vaginal hygiene practises, sexual behaviour and reproductive history were collected. BV was diagnosed by Nugent scoring of Gram-stained vaginal smears. Multivariate regression models were built in order to identify risk factors of BV and to investigate BV as risk factor for adverse pregnancy outcome.**  **The prevalence of BV was 26.3% and approximately half of the women with BV were asymptomatic. Independent risk factors for BV were the use of alternatives to water for intravaginal washing, concurrent partners, the presence of vaginal Candida and clay consumption. BV was independently associated with both low birth weight and preterm delivery with associated low birth weight.** |
| Introduction | | |
| Background/rationale | 2 | Explain the scientific background and rationale for the investigation being reported  **Introduction: Importantly, BV has also been associated with … adverse pregnancy outcomes (APO) such as ….. preterm birth (PTB) and low birth weight (LBW). However, data on the prevalence of BV and risk factors for BV are largely unknown in SSA and – to the best of our knowledge – non-existing for DRC. Furthermore, no studies in DRC, and only very few in SSA, have investigated the association of BV with adverse pregnancy outcomes.** |
| Objectives | 3 | State specific objectives, including any prespecified hypotheses  **Last paragraph of introduction; “…..the aim of this study was to examine the prevalence of BV, the associated risk factors and adverse outcomes in pregnant women from Bukavu, DRC.”** |
| Methods | | |
| Study design | 4 | Present key elements of study design early in the paper  **Methods: a prospective observational study whereby pregnant women were seen between 16 and 20 weeks (visit 1 (V1) = recruitment visit), between 36 and 38 weeks (V2) and at delivery. Newborns were seen at delivery and during the first week of life** |
| Setting | 5 | Describe the setting, locations, and relevant dates, including periods of recruitment, exposure, follow-up, and data collection  **“Hôpital Provincial Général de Référence de Bukavu”, The provincial referral hospital of Bukavu (PRHB) between January and October 2017** |
| Participants | 6 | *(a) Cohort study*—Give the eligibility criteria, and the sources and methods of selection of participants. Describe methods of follow-up  **Methods: Women were considered for inclusion if they were between 16-20 weeks of gestational age, agreed to be followed only by the hospital team, accepted to deliver at PRHB and willing to be contacted by phone. The current study is part of the AVEONS study.** **The AVEONS study was a prospective observational study whereby pregnant women were seen between 16 and 20 weeks (visit 1 (V1) = recruitment visit), between 36 and 38 weeks (V2) and at delivery. Newborns were seen at delivery and during the first week of life.** **All participants followed antenatal care as usual….**  *Case-control study*—Give the eligibility criteria, and the sources and methods of case ascertainment and control selection. Give the rationale for the choice of cases and controls  *Cross-sectional study*—Give the eligibility criteria, and the sources and methods of selection of participants |
|  |  | (*b*) *Cohort study*—For matched studies, give matching criteria and number of exposed and unexposed  *Case-control study*—For matched studies, give matching criteria and the number of controls per case |
| Variables | 7 | Clearly define all outcomes, exposures, predictors, potential confounders, and effect modifiers. Give diagnostic criteria, if applicable  **In methods section outcome, exposure, covariates and confounders are set out in “routine antenatal care and delivery procedures” and “questionnaires” methods sub sections** |
| Data sources/ measurement | 8* | For each variable of interest, give sources of data and details of methods of assessment (measurement). Describe comparability of assessment methods if there is more than one group  **In methods section: At V1, data on the sociodemographic characteristics, reproductive health history, sexual behavior, vaginal practices and complains of the pregnant women were obtained by the obstetrician or the senior assistant in a confidential way by using a questionnaire (Supplementary Information (SI) 1).**  Variables of interest record and measurements (including laboratory results) are summarized in subsection “**Routine antenatal care and delivery procedures**” and “**study specific laboratory tests**” methods subsections. |
| Bias | 9 | Describe any efforts to address potential sources of bias  **In methods section: …In case of discrepancy in categorization, slides were reassessed by the two reviewers and discussed. If no consensus was obtained, a third person assessed the slide as a tie breaker. The raw data were then captured in the CSPRO software by means of double data entry. The ‘compare data tool’ of the CSPRO software was used to compare the two data sets.** |
| Study size | 10 | Explain how the study size was arrived at  **We recruited study participants among pregnant women seeking antenatal care at the Provincial Referral Hospital of Bukavu.. It was a convenience sampling** |
| Quantitative variables | 11 | Explain how quantitative variables were handled in the analyses. If applicable, describe which groupings were chosen and why  **The sample of the smear was then categorized as representing a healthy vaginal microbiome (Nugent score 0-3), an intermediate vaginal microbiome (Nugent score 4-6) or BV (Nugent score 7-10)**  **Neonates were subjected to a general examination and measurement of anthropometric parameters (length, weight and head circumference) by pediatricians. We used World Health Organization definitions for all pregnants outcomes (see Table1 or S1)** |
| Statistical methods | 12 | *(*a) Describe all statistical methods, including those used to control for confounding  **In methods, Categorical variables were summarised into frequencies and proportions, continuous variables into median and interval interquartile range (IQR). A Kappa test was calculated to evaluate agreement in assessing the categorization based on the Nugent score between the two readers of Gram stained slides.** **we built a multivariate model to determine independent risk factors for BV (defined on the basis of a Nugent score of 7-10).** W**e determined whether BV was a risk factor for one or several adverse pregnancy outcomes … A modified Poisson regression model with robust standard error was built using generalized linear regression equations to model the link. Outcome variables with a p-value < 0.05 in the univariate analysis were selected for the multivariate model.** **In this model, we considered vaginal *Candida* colonization (as assessed by means of microscopic examination of Gram-stained slides), anemia, MUAC, cervix length, parity, BMI, maternal age, education level, previous PTB, and/or diastolic high blood pressure at V1 (≥ 90 mm Hg) as possible confounding factors for adverse pregnancy outcomes based on literature** |
|  |  | (*b*) Describe any methods used to examine subgroups and interactions  **No interaction or subgroups has been assessed** |
|  |  | (*c*) Explain how missing data were addressed  **Methods: in STATA 14 (Stata Corp, College Station, Texas, USA)** *the automatically listwise deletion was applied* |
|  |  | (*d*) *Cohort study*—If applicable, explain how loss to follow-up was addressed  *Case-control study*—If applicable, explain how matching of cases and controls was addressed  *Cross-sectional study*—If applicable, describe analytical methods taking account of sampling strategy  **A modified Poisson regression model with robust standard error was built using generalized linear regression equations to model the link.** |
|  |  | (*e*) Describe any sensitivity analyses |

Continued on next page

| Results | | |
| --- | --- | --- |
| Participants | 13* | (a) Report numbers of individuals at each stage of study—eg numbers potentially eligible, examined for eligibility, confirmed eligible, included in the study, completing follow-up, and analysed  **See Figure 1. Flowchart of study** |
|  |  | (b) Give reasons for non-participation at each stage  **See Figure 1. Flowchart of study** |
|  |  | (c) Consider use of a flow diagram  **See Figure 1. Flowchart of study** |
| Descriptive data | 14* | (a) Give characteristics of study participants (eg demographic, clinical, social) and information on exposures and potential confounders  **The sociodemographic characteristics of the study population are outlined in Supplementary Information 1. The median age was 28.0 years (IQR 8.0 years). All participants lived in Bukavu city. The majority (72.6%) lived in poverty, completed the primary school (88.4%) and nearly all (95.5%) were married. Most of the participants were from the Shi tribe (66.7%) and almost all were Christians (93.7%).** |
|  |  | (b) Indicate number of participants with missing data for each variable of interest |
|  |  | (c) *Cohort study*—Summarise follow-up time (eg, average and total amount)  **A total of 533 pregnant women were found eligible and were enrolled in the cohort (V1) ,of which 354 women who completed V2, Of all newborns in the study, 288 (85.5%) were born at term and 49 (14.5%) were born preterm.** |
| Outcome data | 15* | *Cohort study*—Report numbers of outcome events or summary measures over time  **Results, The prevalence of BV, Risk factors for BV and, BV as risk factor for adverse pregnancy outcomes. See Table 1, 2, 3&4 and SI** |
|  |  | *Case-control study—*Report numbers in each exposure category, or summary measures of exposure |
|  |  | *Cross-sectional study—*Report numbers of outcome events or summary measures |
| Main results | 16 | *(*a) Give unadjusted estimates and, if applicable, confounder-adjusted estimates and their precision (eg, 95% confidence interval). Make clear which confounders were adjusted for and why they were included  **See Table 2 for unadjusted estimates and confounder-adjusted estimates for the multivariable model of risk factors associated with bacterial vaginosis in pregnancy. See Table 3 for unadjusted estimates for the analysis of BV and an intermediate vaginal microbiome as risk factor for adverse pregnancy outcomes and confounder-adjusted in Table 3. Analyses were adjusted for vaginal Candida, haemoglobin, Mid-upper arm circumference, cervical length, parity, Body mass index, age, education, previous preterm birth and diastolic blood pressure** |
|  |  | (*b*) Report category boundaries when continuous variables were categorized |
|  |  | (*c*) If relevant, consider translating estimates of relative risk into absolute risk for a meaningful time period **NA** |
| Other analyses | 17 | Report other analyses done—eg analyses of subgroups and interactions, and sensitivity analyses **No sub-set /interactions were tested in the final model** |
| Discussion | | |
| Key results | 18 | Summarise key results with reference to study objectives  **Discussion section, Our study in pregnant women from Bukavu (DRC) shows a BV prevalence of 26.3%. To the best of our knowledge no studies on BV have been performed in DRC. In our study, nearly half of women diagnosed with BV by means of the Nugent score were asymptomatic. We found that pregnant women whose partners had concurrent sexual partners had a two-fold increase in BV compared to pregnant women whose partner did not have other sexual partners.** **We found that women who applied intravaginally substances such as soap, antiseptics, lemon juice or herbs had a nearly two-fold higher chance of having BV.** **In our study, concurrent vaginal *Candida* colonization was independently associated with an approximately two-fold increased odds for BV.** **This geophagia was independently associated with a lower chance of having BV.** **Pregnant women with BV had a threefold increased risk to deliver a neonate with LBW and a fourfold increased risk to deliver a preterm neonate with LBW.** |
| Limitations | 19 | Discuss limitations of the study, taking into account sources of potential bias or imprecision. Discuss both direction and magnitude of any potential bias  **Discussion section, our study was limited by the fact that the number of women who delivered at our hospital maternity was less than had been foreseen due to the socio-political situation in Bukavu during the study period. Also, we only analysed BV in second trimester and not first and third trimester.** |
| Interpretation | 20 | Give a cautious overall interpretation of results considering objectives, limitations, multiplicity of analyses, results from similar studies, and other relevant evidence  **Discussion section, last paragraph, BV was highly prevalent in pregnant women in Bukavu, and associated with modifiable risk factors such as vaginal hygiene practices. Given the well-established knowledge that BV constitutes an increased risk for important adverse pregnancy outcomes, further local research is needed to better understand this link and to reduce BV prevalence in pregnancy.** |
| Generalisability | 21 | Discuss the generalisability (external validity) of the study results  **Discussion the last paragraph: “In conclusion, BV was highly prevalent in pregnant women in Bukavu, and associated with modifiable risk factors such as vaginal hygiene practices.”** |
| Other information | | |
| Funding | 22 | Give the source of funding and the role of the funders for the present study and, if applicable, for the original study on which the present article is based  **This study was funded by the Flemish government (VLIR-UOS)** |

*Give information separately for cases and controls in case-control studies and, if applicable, for exposed and unexposed groups in cohort and cross-sectional studies.

**Note:** An Explanation and Elaboration article discusses each checklist item and gives methodological background and published examples of transparent reporting. The STROBE checklist is best used in conjunction with this article (freely available on the Web sites of PLoS Medicine at http://www.plosmedicine.org/, Annals of Internal Medicine at http://www.annals.org/, and Epidemiology at http://www.epidem.com/). Information on the STROBE Initiative is available at www.strobe-statement.org.
